# Distinctive Patterns of Transcription and RNA Processing for Human lincRNAs

**DOI:** 10.1016/j.molcel.2016.11.029

**Published:** 2017-01-05

**Authors:** Margarita Schlackow, Takayuki Nojima, Tomas Gomes, Ashish Dhir, Maria Carmo-Fonseca, Nick J. Proudfoot

**Affiliations:** 1 Sir William Dunn School of Pathology, University of Oxford, South Parks Road, Oxford OX1 3RE, UK; 2 Instituto de Medicina Molecular, Faculdade de Medicina, Universidade de Lisboa, 1649-028 Lisboa, Portugal

**Keywords:** mNET-seq, lincRNA, splicing, phosphor CTD marks, exosome, transcription termination, polyadenylation, CPSF73, empigen

## Abstract

Numerous long intervening noncoding RNAs (lincRNAs) are generated from the mammalian genome by RNA polymerase II (Pol II) transcription. Although multiple functions have been ascribed to lincRNAs, their synthesis and turnover remain poorly characterized. Here, we define systematic differences in transcription and RNA processing between protein-coding and lincRNA genes in human HeLa cells. This is based on a range of nascent transcriptomic approaches applied to different nuclear fractions, including mammalian native elongating transcript sequencing (mNET-seq). Notably, mNET-seq patterns specific for different Pol II CTD phosphorylation states reveal weak co-transcriptional splicing and poly(A) signal-independent Pol II termination of lincRNAs as compared to pre-mRNAs. In addition, lincRNAs are mostly restricted to chromatin, since they are rapidly degraded by the RNA exosome. We also show that a lincRNA-specific co-transcriptional RNA cleavage mechanism acts to induce premature termination. In effect, functional lincRNAs must escape from this targeted nuclear surveillance process.

## Highlights

• lincRNAs and pre-mRNAs are transcribed by different Pol II phospho-CTD isoforms
• lincRNAs are rarely spliced and mainly non-polyadenylated
• lincRNAs are stabilized in the nucleoplasm following exosome inactivation
• lincRNAs are co-transcriptionally cleaved

## Graphical Abstract

**Figure:**
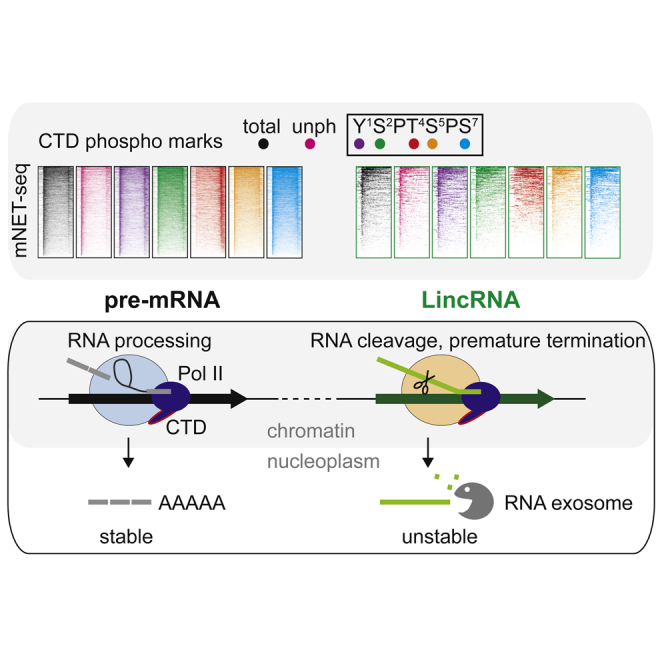

## Teaser

Schlackow and Nojima et al. show that mammalian pre-mRNAs and long intergenic noncoding (linc) RNAs employ radically different transcription and RNA-processing strategies. Pre-mRNAs are transcribed by defined RNA polymerase (Pol) II isoforms reflecting co-transcriptional splicing and polyadenylation. Instead, lincRNAs are mainly transcribed by deregulated Pol II and simultaneously degraded.

## Introduction

Approximately 20,000 protein-coding genes are transcribed by RNA polymerase II (Pol II) from the human genome. These transcripts are modified by pre-mRNA processing events, such as 5′ capping, pre-mRNA splicing, 3′ end cleavage, and polyadenylation during Pol II transcription (Moore and Proudfoot, 2009). Pre-mRNA processing as well as generating translatable mature mRNA also acts to enhance mRNA stability and cytoplasmic export. Even though protein-coding genes occupy a limited proportion of the mammalian genome, transcription analyses reveal the widespread occurrence of long noncoding RNAs (lncRNAs), which lack significant protein-coding capacity (St Laurent et al., 2015). In general, lncRNA can be subdivided into different classes based on their positional relationship to protein-coding transcripts. Thus, Pol II promoters as well as generating pre-mRNAs also form promoter upstream transcripts in antisense orientation, called CUTs in *S. cerevisiae* or PROMPTs in mammals (Jensen et al., 2013). Additionally, in higher eukaryotes, multiple Pol II enhancers exist upstream or within protein-coding genes that act to guide Pol II to promoters by *trans* interactions. These numerous enhancers also generate bidirectional transcripts called eRNAs (Kim et al., 2010, Kowalczyk et al., 2012). Finally, some lncRNA initiate independently of protein-coding gene promoters and enhancers to generate separate transcription units (TUs) called long intervening noncoding RNA (Ulitsky and Bartel, 2013). It is the focus of this study to better understand how long intervening noncoding RNAs (lincRNAs) are synthesized and processed and how this may differ from protein-coding genes.

Whereas PROMPTs and enhancer RNAs (eRNAs) likely form as a consequence of Pol II accumulation at transcription initiation sites, it is more plausible that lincRNAs, with their independently defined transcription units, have specific biological significance. However, their low sequence conservation and often very low steady-state levels imply that many of these ephemeral transcripts reflect transcriptional noise (Struhl, 2007). One often proposed argument for lincRNA functionality is that they are at least partially capped, spliced, and polyadenylated, based on high-throughput cDNA analysis. This has led to the view that lincRNAs are mRNA like (Cabili et al., 2011, Derrien et al., 2012, Garber et al., 2011, Grabherr et al., 2011). Although the function of most lincRNAs remains unknown, some, such as XIST, HOTAIR, NORAD, and FENDRR, have established biological roles (Grote et al., 2013, Lee et al., 2016, Mattick, 2009, St Laurent et al., 2015, Wang and Chang, 2011).

Defining the TUs of lincRNAs is a challenging problem of sequence annotation. Often transcription start sites are inferred from 5′ end cap selection methods, such as CAGE (Kodzius et al., 2006) or Cap-seq (Gu et al., 2012). However, some degree of recapping has been shown to occur on cytoplasmic RNA (Affymetrix ENCODE Transcriptome Project and Cold Spring Harbor Laboratory ENCODE Transcriptome Project, 2009), so that some capped lincRNAs may derive from RNA degradation intermediates. Also a recent description of chromatin-associated lncRNAs included many cases of low-level readthrough transcription from upstream protein-coding gene TUs (Werner and Ruthenburg, 2015). The realization that cellular stress can increase readthrough transcription for protein-coding genes (Vilborg et al., 2015) may exacerbate such problems of mis-annotation. For lincRNAs, 3′ end mapping by poly(A) selection methods are often employed, such as the 3P-seq method (Ulitsky and Bartel, 2013). Such approaches may not be appropriate for lincRNAs, as these transcripts are often unpolyadenylated, such as those harboring pre-microRNA (miRNA) sequences (Dhir et al., 2015). Also, lincRNA 3′ ends may be subject to rapid 3′ end degradation by the nuclear exosome (Pefanis et al., 2015, Lubas et al., 2015). Finally, previous annotations of lincRNAs have focused on spliced transcripts as a way to increase specificity. However, we show that lincRNAs are generally only weakly spliced and so may be excluded from such analysis (Cabili et al., 2011). Indeed, transcription regulation of lincRNA genes remains poorly characterized due to a lack of detailed information on how they are synthesized and processed.

Recently, we have developed mammalian native elongating transcript sequencing (mNET-seq) to precisely define nascent transcription across the human genome (Nojima et al., 2015). In particular, we have focused on the C-terminal domain (CTD) of the largest subunit of Pol II, which has a 52 times repeated heptad domain (Y_1_S_2_P_3_T_4_S_5_P_6_S_7_) that is differentially phosphorylated during Pol II transcription (Heidemann et al., 2013). mNET-seq allows the determination of which CTD phosphorylation marks correlate with different stages of TU synthesis and processing. Here, we obtained mNET-seq profiles using a full range of Pol II CTD antibodies to compare the expression profiles between protein-coding and lincRNA TUs. We show that most lincRNAs, unlike protein-coding genes, are poorly co-transcriptionally spliced, and Pol II pauses inefficiently at their promoters. Furthermore, the CTD T4P mark that correlates with protein-coding gene termination is distributed more evenly across the gene body of lincRNAs. This implies that lincRNA termination occurs at multiple positions within the TU. Also, mRNA 3′ end processing endonuclease CPSF73 shows little effect on lincRNA 3′ end formation. These observations in general indicate that lincRNA and pre-mRNA processing differ both quantitatively and qualitatively.

## Results

Widespread lincRNAs have been defined in several comprehensive studies (Ulitsky and Bartel, 2013). Although combined transcription profiles from multiple cell types show that most human intergenic sequences (regions between annotated protein coding genes) are transcribed, within one specific cell type, lincRNA expression is more restricted. We have analyzed lincRNA expression in human HeLa cells where about 35% of the non-repetitive genome is transcriptionally active (Djebali et al., 2012). Of roughly 50,000 annotated Tus, about 20,000 are protein coding. To define the gene units of expressed lincRNAs for our analyses, we employed ENSEMBL and NONCODE databases as reference gene annotation (Flicek et al., 2014, Xie et al., 2014). We then cross-checked these annotations by visual identification of their transcription start and end sites (TSSs and TESs) using our own HeLa cell RNA sequencing (RNA-seq) data from chromatin and nucleoplasm fractions (Nojima et al., 2015). We excluded low-level expressed lincRNAs as well as lincRNAs that were close to other TUs either at their TSSs or TESs, including those annotated as an antisense biotype in the ENSEMBL annotation. This generated a list of 285 lincRNAs that are expressed at sufficiently high levels separate from other adjacent transcription units to allow their independent analysis (Tables S1 and S2). In the later stages of this study, we included the antisense biotype to effectively add 500 additional lincRNAs (antisense RNAs).

### Pol II CTD Phosphorylation Profiles Differ between Pre-mRNAs and lincRNAs

**Figure 1:**
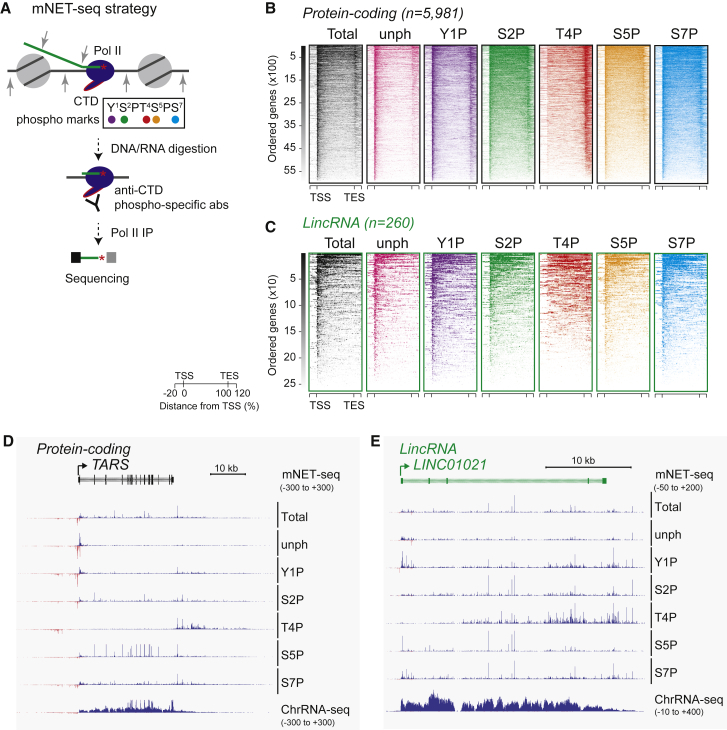
Differential mNET-Seq Profiles for Protein Coding and lincRNA genes (A) mNET-seq strategy with each Pol II phospho-CTD modification color coded. (B and C) Color-coded heatmaps showing phospho-CTD profiles across individual (B) protein coding TUs and (C) lincRNA TUs ordered based on their transcription levels. Profiles are aligned to TSS and TES as indicated. Genes >1,000 nt (excluding some smaller protein coding and lincRNA genes) were divided into 100 bins. (D and E) (D) mNET-seq profiles across *TARS* (black for protein-coding gene) and (E) *LINC01021* (green for lincRNA gene) using seven different Pol II antibodies as indicated. Gene maps show exons filled in and introns hatched. A chromatin-seq profile is run below the mNET-seq profiles. Blue reads are sense and red reads antisense transcripts. Reads per 10^8^ mapped reads are indicated in brackets. See also Figure S1.

Pol II CTD phosphorylation states are well established to match different transcriptional stages: Ser5P (S5P) with early elongation, 5′ end capping, and active splicing and Ser2P (S2P) with later elongation and 3′ end processing (Heidemann et al., 2013, Hsin and Manley, 2012). mNET-seq methodology sequences genome-wide nascent RNA at single nucleotide resolution (Nojima et al., 2015) by isolating RNA from immunoprecipitated (IP) Pol II. We previously employed Pol II antibodies against total, S2P, S5P, and unphosphorylated (unph) CTD to isolate specific nascent RNA fractions (Nojima et al., 2015). Here, we have added three additional phospho-CTD-specific antibodies, Y1P, T4P, and S7P, allowing a closer comparison between protein-coding and lincRNA genes (Figure 1A).

Meta-analysis of protein coding as compared to lincRNA genes reveals significant differences in mNET-seq profiles. Both heatmaps and metagene profiles (Figures 1B, 1C, and S1A) are shown. In particular, the unph followed by Y1P profiles show highest promoter peaks for protein-coding genes. In contrast, lincRNA genes show less pronounced unph and Y1P TSS peaks with a generally more even distribution of mNET-seq reads across their gene bodies. A wider set of lincRNA TUs that are partly overlapping with other TUs (ENSEMBL antisense biotype) looks closely similar to the separate lincRNA TU class (Figure S1A). We also included analysis of TSS-associated eRNAs (both strands), which derive from unph Pol II with some from Y1P Pol II, but very little with other phospho-CTD isoforms (Figure S1A, bottom panel). We next compared the promoter escape indexes between protein coding and lincRNA genes, taken as the ratio of reads in TSS regions versus gene body. Lower Pol II pausing was observed over the TSS regions of lincRNA than protein-coding genes, as shown in data replicates (Figure S1B, top; p < 1e−5 for unph [both replicates] and p < 1e−6 for Y1P [all three replicates]).

A notable feature of the TES region in protein-coding genes is the high T4P signal, which is indicative of Pol II termination (Figure 1B). This observation is consistent with previous chromatin immunoprecipitation sequencing (ChIP-seq) results (Hintermair et al., 2012). In contrast, T4P signal over lincRNA genes is more evenly distributed across the whole TU, with less TES-associated accumulation (Figures 1C and S1A), suggesting that Pol II termination occurs at multiple positions across lincRNA TUs. These replicated TES effects were quantitated by their termination indices, which are taken as the ratio of reads in termination regions versus gene body (Figure S1B, bottom). We observe a lower T4P termination index in lincRNA compared to coding genes (p < 1e–10; all three replicates). The metagene analysis is consistent with individual gene profiles of mNET-seq for the protein-coding gene *TARS* and a specific lincRNA gene (Figures 1D and 1E). Overall, mNET-seq reveals significant differences in Pol II CTD phosphorylation between protein-coding and lincRNA genes.

### lincRNAs Are Inefficiently Spliced

**Figure 2:**
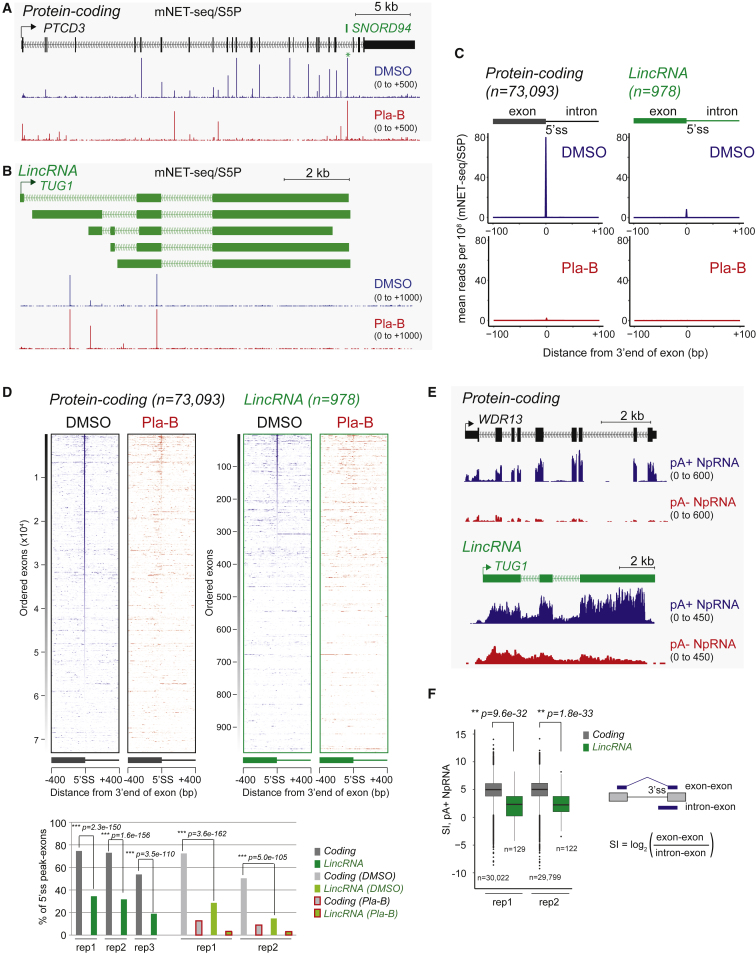
lincRNAs Are Inefficiently Spliced (A and B) (A) mNET-seq/S5P analysis of protein-coding gene *PTCD3* and (B) lincRNA *TUG1*. HeLa cells were treated with Pla-B (red) or DMSO control (blue). Only sense transcripts are shown. (C) Meta-analysis across exon-intron junctions (5′ss) of annotated introns for protein-coding TUs versus lincRNAs. (D) Heatmaps for protein-coding versus lincRNA genes aligned to 5′ss −400 to +400 nt upstream and downstream. Percent of introns showing co-transcriptional 5′ss peaks is shown below, including all data repetitions, either with untreated, DMSO mock-treated, or Pla-B-treated HeLa cells. (E) pA+ and pA− NpRNA-seq profiles are shown for *WDR13* versus lincRNA *TUG1*. (F) Splicing index from pA+ NpRNA-seq for protein-coding and lincRNA TUs (duplicates shown). See also Figure S2.

We have previously identified a characteristic mNET-seq pattern associated with co-transcriptional splicing. In particular, a prominent splicing intermediate derived from RNA cleavage at 5′ splice sites (5′ss) is evident in mNET-seq/S5P profiles of protein-coding genes (Nojima et al., 2015), as seen for the multi-intronic protein-coding gene *TARS* (Figure 1D). These 5′ss peaks are indicative of co-transcriptional splicing, where upstream exons are tethered to Pol II S5P CTD prior to splicing with the downstream exon to complete the splicing reaction. mNET-seq/S5P also detects several peaks on the lincRNA gene *LINC01021*. However, these were not S5P CTD specific, showing similar patterns for S7P and S2P analysis, nor were they exon specific, appearing to derive from intronic regions (Figure 1E). We next extended our analysis of specific lincRNAs using splicing specific mNET-seq/S5P profiles and tested their sensitivity to pretreatment of the HeLa cells with the chemical inhibitor Pla-B. This blocks splicing by direct binding to the SF3B complex (Kotake et al., 2007). As previously reported (Nojima et al., 2015), Pla-B erased most of the S5P CTD-specific 5′ss peaks on protein-coding genes as shown for *PTCD3* (Figure 2A). This confirms that these peaks derive from an active splicing process. Notably, a few *PTCD3* intronic peaks were either unaffected or enhanced by Pla-B treatment. These may reflect the maturation of small RNAs from intronic locations, such as *SNORD94*. In contrast, Pla-B treatment had a more limited effect on S5P peaks seen across various lincRNA genes (Figures 2B and S2A). Indeed, only two Pla-B-sensitive splicing events were detectible for these specific lincRNAs: 5′ss of *LINC00472* intron 3 and *LINC00263* intron 1.

To establish generality for lower co-transcriptional splicing on lincRNAs, we obtained mNET-seq/S5P meta-analysis profiles across the exon-intron boundaries of about 70,000 annotated introns for protein coding versus 1,000 for lincRNA genes with or without Pla-B treatment. Both average signals and heatmaps (Figures 2C and 2D) of the whole dataset show Pla-B-sensitive 5′ss signals occur less frequently for lincRNA than protein-coding genes. Quantitation of these data in all biological replicas indicates that 55%–70% of protein-coding introns give 5′ss peaks. Possibly those that lack detectible peaks reflect unspliced exons due to alternative splicing events or retained introns (Boutz et al., 2015). In contrast, only 20%–30% of lincRNA exons gave 5′ss peaks, reflecting lower levels of co-transcriptional splicing (Figure 2D, bottom).

The above data focus on the levels of co-transcriptional splicing based on 5′ss mNET-seq/S5P signals and clearly indicates reduced lincRNA co-transcriptional splicing. To directly measure splicing efficiency, we prepared duplicate HeLa cell transcript libraries from either pA+ or pA− nuclear RNA. pA+ reads across the specific protein-coding gene *WDR13* were exon restricted, indicative of efficient co-transcriptional spicing with little signal detected in the pA− NpRNA-seq profile. In contrast, for the lincRNA *TUG-1* pA+ profile, significant levels of intron reads were detected over its annotated intron regions, even though some splicing is evident. Furthermore, the pA− profile revealed a higher level of intron signal (Figure 2E). We performed quantitative analysis of splicing efficiency between protein coding and lincRNA transcripts. Comparison of splicing events between these two transcript classes for pA+, pA−, and total nucleoplasmic RNA showed a consistently lower splicing for lincRNAs in duplicate experiments (Figure S2B). We finally computed the splicing index of protein coding versus lincRNA by comparing the ratios of spliced exon-exon to unspliced intron-exon reads across active 3′ss in NpRNA-seq, either pA+, pA−, or total (Figures 2F and S2C). This quantitation reveals that lincRNA are inefficiently spliced as compared to protein-coding genes. Note that the duplicated pA+ and pA− NpRNA-seq analyses were closely consistent (Figure S2D).

### lincRNAs Are Inefficiently Polyadenylated

Our mNET-seq/T4P datasets show a close correlation between the CTD T4P mark and protein-coding gene termination (Figure 1). In contrast, lincRNAs show reduced T4P 3′ end association, with many showing a more widespread T4P profile across the whole TU. We previously demonstrated, based on mNET-seq/S2P analysis, that Pol II pauses over the 3′ end of protein-coding genes in a cleavage and polyadenylation factor (CPA)-dependent manner (Nojima et al., 2015). Thus, RNAi depletion of either CPSF73, the CPA endonuclease, or CstF-64/64tau, which recognize pA signal (PAS) downstream regions, markedly reduces this pausing effect.

**Figure 3:**
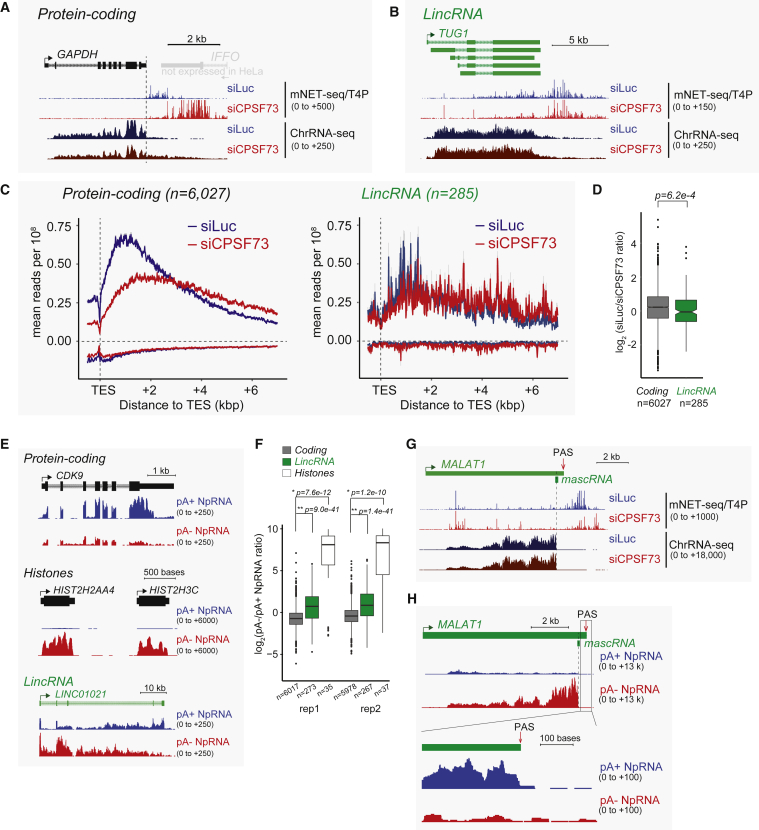
lincRNAs Are Largely Unpolyadenylated and CPA Independent (A and B) (A) mNET-seq/T4P analysis of *GAPDH* and (B) lincRNA *TUG1*. Vertical dotted line over *GAPDH* denotes PAS. (C) Meta-analysis of termination region (up to 7 kbp 3′ to TES) associated mNET-seq/T4P profiles, ±CPSF73 depletion by small interfering RNA (siRNA) treatment. siLuc indicates siRNA control treatment. Protein-coding TUs are shown on the left and lincRNA TUs on the right. (D) Quantitation of readthrough transcript levels following CPSF73 depletion characterized by GB-signal-normalized siLuc to siCPSF73 signal ratio in 10 kbp downstream of TES. (E) Gene-specific profiles (*CDK9*, histone *H2A*, histone *H3*, and *LINC01021*) for pA+ and pA−NpRNA-seq. (F) Quantitation of levels of pA−/pA+ transcripts for protein coding versus lincRNA TUs based on number of fragments overlapping TUs. Duplicate data are shown. (G) mNET-seq/T4P versus ChrRNA-seq profiles for *MALAT1*. mascRNA and PAS positions are indicated. (H) pA+/pA− RNA-seq for MALAT1. 3′ end of TU is expanded. See also Figure S3.

We extended our previous data by testing the effect of CPSF73 depletion (Figure S3A) on mNET-seq/T4P profiles in duplicate. First, the specific patterns obtained for *GAPDH* versus *TUG-1* underlie the differences generally seen for protein coding versus lincRNA genes. Thus, *GAPDH* shows a clear accumulation of mNET-seq reads over the termination region that substantially shifts downstream following CPSF73 depletion (Figure 3A). Even though *GAPDH* shows a loss of PAS-dependent termination following CPSF73 depletion, a further downstream termination region is evident based on an abrupt loss of mNET-seq/T4P reads at a downstream position. We generally see this effect for protein-coding genes (Figure S3B), which may reflect a CPA-independent fail-safe termination process. Whereas the lincRNA *TUG1* profile for mNET-seq/T4P also detects some 3′ end peaks, depletion of CPSF73 does not affect this profile, suggesting *TUG1* termination is CPSF73 independent (Figure 3B). Four other lincRNAs gave similar results (Figure S3C), although *LINC00052* displayed some CPA-dependent termination especially visible in the ChrRNA-seq profiles. Again, we performed meta-analyses on the duplicate databases (Figures 3C and S3D), showing that protein coding, but not lincRNA gene termination, is strongly affected by CPSF73 depletion. We finally quantitated the effect of CPSF73 depletion on TES pausing and show that there is a significant effect on protein-coding genes compared to lincRNAs (p = 6.2e−4; Figure 3D).

To examine the degree of 3′ end polyadenylation in lincRNAs, we again employed our pA+ and pA− NpRNA-seq libraries. As expected, protein-coding transcripts were predominantly pA+, as exemplified by the *CDK9* gene (Figure 3E, top). In contrast, histone RNAs were exclusively in the pA− fraction (Figure 3E, middle), because histone mRNA is maturated by a PAS-independent mechanism (Dominski and Marzluff, 2007). Notably, lincRNAs, such as *LINC01021*, display higher pA− than pA+ reads (Figure 3E, bottom). In general, lincRNAs are inefficiently polyadenylated as compared to protein-coding transcripts as shown in our duplicated experiments (Figure 3F).

We also investigated the mNET-seq and ChrRNA-seq profiles of the lincRNA *MALAT1*. This lincRNA lacks a pA tail, being processed by RNase P to generate a 3′ terminal tRNA-like RNA, known as *MALAT1*-associated small cytoplasmic RNA (mascRNA) (Wilusz et al., 2008). The upstream *MALAT1* RNA is stabilized by the formation of a 3′ terminal triple helical structure (Brown et al., 2014). Notably, mNET-seq/T4P-detected reads peak at a TES position several kilobases downstream of mascRNA. Interestingly, this pause region is decreased by CPSF73 knockdown, suggesting *MALAT1* termination is CPA dependent (Figure 3G). Consistent with this possibility, a PAS is known to be present at the end of this downstream region (Wilusz et al., 2008). Whereas *MALAT1* is mainly present in the pA− nucleoplasm RNA fraction due to RNase P cleavage, a small fraction of *MALAT1* RNA extending beyond the RNase P site to the PAS was detected in the pA+ fraction (Figure 3H). We also analyzed mNET-seq/S2P profiles for *MALAT1*, showing a clear termination defect following CstF64/64tau depletion (Nojima et al., 2015; Figure S3E). Furthermore, these CPA factors crosslink to the *MALAT1* PAS region based on PAR-CLIP analysis (Martin et al., 2012). Overall, these results imply a kinetic model for *MALAT1* 3′ end processing, where Pol II termination is mediated by the CPA complex at a downstream PAS, followed by co- or post-transcriptional RNase P cleavage in the nucleoplasm.

### lincRNAs Are Degraded Post-transcriptionally by the Nuclear Exosome

**Figure 4:**
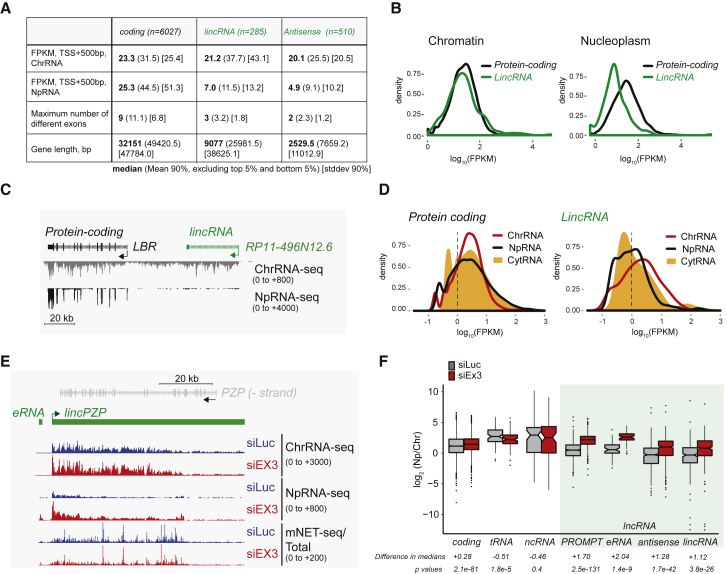
lincRNAs Are Chromatin Restricted and Degraded by the Nuclear Exosome (A) Transcription levels for coding, lincRNA, and antisense RNA in chromatin or nucleoplasm as well as exon numbers and gene lengths. (B) Density plots of chromatin and nucleoplasm fragments per kilobase of transcript per million of mapped reads (FPKM) levels (first 500 bp) for protein-coding and lincRNA TUs. (C) ChrRNA-seq versus NpRNA-seq for tandem lincRNA and *LBR* locus. (D) Density plots of FPKM levels in chromatin, nucleoplasm, and cytoplasm comparing protein-coding and lincRNA TUs. (E) Comparison of ChrRNA-seq, Np-RNA seq, and mNET-seq/total Pol II for *lincPZP* ± exosome (EXOSC3). *lincPZP* is antisense to the protein-coding gene *PZP* (not expressed in HeLa cells). (F) Quantitation of ratios of nucleoplasm to chromatin RNA levels for different classes of transcript as indicated. Non-coding RNA (ncRNA) denotes stable RNA, such as snRNA and snoRNA. See also Figure S4.

Even though some lincRNAs have been reported to be functional (Quinn and Chang, 2016), we show above that this transcript class is both poorly spliced and polyadenylated (Figures 2 and 3). This led us to a study of lincRNA stability. We initially compared the levels of transcript reads over the TSS regions of protein coding versus lincRNA and also the antisense lncRNA class (Table S2). As shown (Figures 4A and 4B), whereas lincRNA and protein-coding gene transcripts are often similar in abundance in the chromatin fraction, lincRNA levels are substantially reduced in the nucleoplasm. In particular, we show transcription profiles for a tandem lincRNA and protein-coding gene *LBR* (Figure 4C). Whereas ChRNA-seq read levels are similar across these two adjacent TUs, little lincRNA is detectable in the nucleoplasm, suggesting that it is degraded post-transcriptionally. We also interrogated published RNA-seq data (Mayer et al., 2015) for lincRNA expression in the cytoplasm to exclude the possibility of rapid nuclear export. Again, much less cytoplasmic lincRNA is detected as compared to chromatin-associated lincRNA (Figure 4D).

It has been previously established that lncRNAs are substrates of the RNA exosome in mouse embryonic stem cells (ESCs) (Pefanis et al., 2015). However, in this study, total cellular RNA was analyzed so that it was not determined where in the cell such RNA degradation occurs. Exosome-mediated degradation of lncRNA may be triggered by the nuclear complex NEXT, which acts as an adaptor to recruit exosome to susceptible capped Pol II transcripts (Andersen et al., 2013, Lubas et al., 2015). We therefore depleted the RNA exosome component EXOSC3 (Figure S4A), which is essential for exosome activity (Chlebowski et al., 2013), and performed duplicate ChrRNA-seq and NpRNA-seq. Interestingly, lincRNAs were all significantly increased in the nucleoplasm by EXOSC3 knockdown, although RNA levels in chromatin (both ChRNA-seq and mNET-seq) were unaffected (Figures 4E and S4B). We also compared the ratio of chromatin to nucleoplasm RNA levels between protein-coding and definable classes of lncRNA genes following exosome depletion (Figures 4F and S4C). Notably, protein-coding RNA levels (first 500 nt) were slightly stabilized, suggesting some low-level turnover by the exosome of possibly mis-spliced mRNAs (Davidson et al., 2012). In contrast, tRNAs and structural ncRNAs (such as small nuclear RNAs [snRNAs]) were significantly destabilized by exosome inactivation, consistent with the known role of the exosome in tRNA and snRNA maturation (Schneider et al., 2012). Remarkably, all categories of lncRNAs (PROMPTs, eRNAs, antisense RNAs, and lincRNAs) show significant nucleoplasmic stabilization following exosome depletion. Because EXOSC3 depletion does not affect mNET-seq profiles (Figures 4E and S4B), we conclude that lincRNAs are downregulated by the nuclear RNA exosome in the nucleoplasm (Figure S4D).

### Co-transcriptional RNA Cleavage of lincRNAs

We predict from the widespread profiles of mNET-seq/T4P reads across lincRNA TUs that Pol II terminates sporadically across this gene class (Figure 1). Additionally, the nuclear exosome degrades lincRNAs post-transcriptionally (Figure 4). These observations lead to the hypothesis that co-transcriptional RNA cleavage activity acting on lincRNAs might induce premature termination and that the cleaved RNA so formed can then act as a substrate for the nuclear exosome. To investigate this possibility, we searched for evidence of co-transcriptional RNA cleavage activity in our mNET-seq profiles.

**Figure 5:**
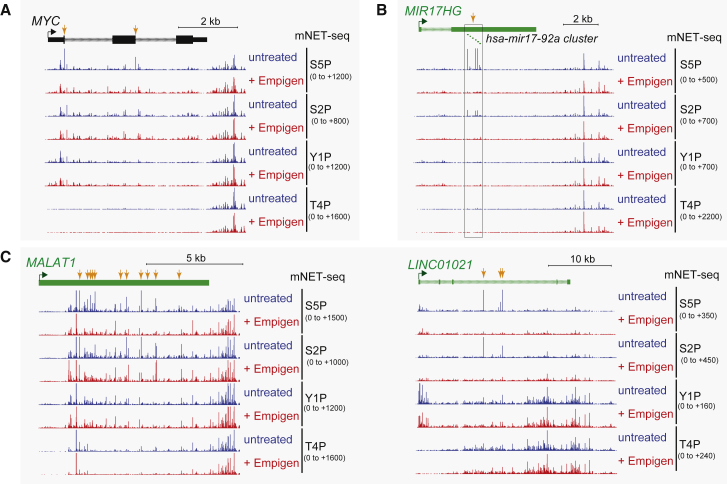
Identification of Co-associated RNA-Processing Complexes with Pol II Comparison mNET-seq/S5P, S2P, Y1P, and T4P profiles with or without Empigen treatment for (A) *MYC*, (B) *MIR17HG*, (C) *MALAT1*, and (C) *LINC01021*, respectively. Orange arrows denote Empigen-sensitive peaks. See also Figure S5.

The mNET-seq technique involves the ligation of a linker oligonucleotide onto any RNA 3′ end protected from micrococcal nuclease digestion. These principally derive from the Pol II active site, reflecting nascent transcription. However, co-precipitated RNA processing complexes, such as the spliceosome or microprocessor, can also generate RNA 3′ ends (detected by mNET-seq), such as splicing intermediates or microRNA precursors (Nojima et al., 2015). Because the positions of such RNA cleavage intermediates are well known (i.e., 5′ splice sites or pre-microRNA Drosha cleavage sites), their identification proved straightforward. However, RNA 3′ ends formed by unidentified RNA-processing complexes may also be co-precipitated with Pol II. To separate mNET-seq reads derived from Pol II active site RNA 3′ ends and those derived from co-precipitated RNA processing complexes, we employed the detergent Empigen to separate the Pol II core machinery from Pol-II-associated complexes, such as the spliceosome and microprocessor. Empigen is known to weaken many protein-protein interactions, but not high-affinity antigen-antibody interactions (Choi and Dreyfuss, 1984), suggesting that strong interactions should be resistant to Empigen treatment. We therefore added Empigen to the Pol II IP step in the mNET-seq procedure. As shown for mNET-seq analysis of the *MYC* gene, S5P-specific 5′ss peaks are specifically lost with Empigen treatment, presumably because the co-immunoprecipitated spliceosome containing this splicing intermediate is now released from the Pol II complex (Figure 5A). This was confirmed for a specific protein component of the spliceosome (U5 116k; Figure S5A). Similarly, the S5P-/S2P-specific microprocessor-mediated RNA cleavage intermediate is lost from the lincRNA *MIR17HG* following Empigen treatment (Figure 5B). Importantly, Y1P and T4P CTD mNET-seq signals were unaffected by Empigen treatment, implying that they all derive from the Pol II active site (Figures 5A and 5B). In addition, other signals, such as TSS-associated peaks, were unaffected (data not shown). All Empigen-treated mNET-seq libraries were duplicated and show highly consistent profiles.

Our mNET-seq analysis of individual lincRNAs, unlike protein-coding genes, reveals numerous Empigen-sensitive peaks, as shown for *MALAT1* and *LINC01021* in mNET-seq/S5P and S2P profiles (Figure 5C) and several other lincRNAs (Figure S5B). In many cases, peak levels reduced rather than completely disappeared. These Empigen-sensitive peaks indicate that lincRNAs are co-transcriptionally cleaved at multiple positions across their TUs. Notably, most Empigen-sensitive lincRNA peaks are insensitive to Pla-B treatment (Figure S2A), indicating that they are distinct from splicing intermediates (Nojima et al., 2015). Overall, we show that Empigen treatment can be employed to distinguish co-transcriptional RNA cleavage activity from ongoing transcription in the Pol II active site.

### Role of RNAi Factors in lincRNA Degradation

**Figure 6:**
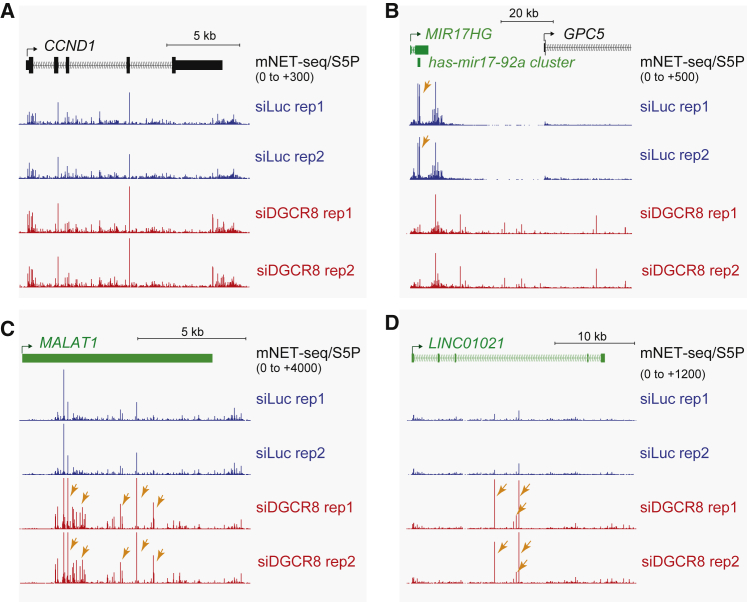
Effect of DGCR8 Depletion on Co-transcriptional Processing mNET-seq/S5P profiles for (A) *CCND1*, (B) *MIR17HG-GPC5*, (C) *MALAT1*, and (D) *LINC01021* with DGCR8 siRNA-mediated depletion or control siLuc treatment. Orange arrow indicates loss of pre-miRNA cleavage for *MIR17HG* or elevated levels of cleavage products following DGCR8 depletion for *MALATI* and *LINC01021*. Duplicate mNET-seq/S5Ps are presented to underline data reproducibility. See also Figure S6.

We reasoned that possible endonucleases responsible for lincRNA degradation could be either nuclear Drosha as part of the microprocessor (with DGCR8) or the related RNase III endonuclease Dicer. Although Dicer activity is predominantly cytoplasmic, where it acts to process pre-microRNA into microRNA (Ha and Kim, 2014), nuclear Dicer has been reported in recent studies to play various roles in nuclear RNAi pathways (Burger and Gullerova, 2015). We therefore generated mNET/S5P datasets using chromatin from HeLa cells depleted for either DGCR8 or Dicer (Figure S6A). Note that DGCR8 depletion also inactivates Drosha as an integral part of the microprocessor (Dhir et al., 2015). Neither DGCR8 nor Dicer depletion affected mNET-seq/S5P profiles on the protein-coding gene *CCND1* (Figures 6A and S6B). In contrast, for *MIR17HG*, which encodes the miR17-92a cluster, mNET-seq peaks corresponding to release of these pre-miRNAs were abolished and a transcription termination defect was detected (Figures 6B and S6C) following DGCR8, but not Dicer, depletion. This confirms that microprocessor-mediated cleavage of linc-pre-miRNAs induces Pol II termination defects (Dhir et al., 2015). However, neither loss of the microprocessor (by DGCR8 knockdown) nor Dicer caused a general loss of lincRNA mNET-seq/S5P peaks (Figures 6C, 6D, S6D, and S6E), arguing against a role for these endonucleases in lincRNA cleavage.

Recent studies show that DGCR8, the RNA-binding component of the microprocessor, interacts with nuclear RNA exosome components, independently of the endonuclease Drosha (Macias et al., 2015). In this situation, it facilitates exosome recruitment to degrade abundant lncRNAs, such as small nucleolar RNAs (snoRNAs) and human telomerase RNA component (hTERC). Because we show that the nuclear RNA exosome degrades lincRNAs, we investigated whether DGCR8 is also involved in lincRNA turnover. Interestingly, DGCR8, but not Dicer, depletion acted to selectively increase Empigen-sensitive mNET-seq/S5P peaks on lincRNA genes, such as *MALAT1* and *LINC01021* (Figures 6C and 6D). This suggests that DGCR8 also acts to recruit the exosome to co-transcriptionally cleaved lincRNA, independently of miRNA. Consistent with our mNET-seq data, some lincRNA levels increase at a steady-state level based on whole-cell RNA-seq analysis (Figure S5C; Macias et al., 2015).

### PCA Reveals lincRNAs Are Generally Distinct from Protein-Coding Genes

**Figure 7:**
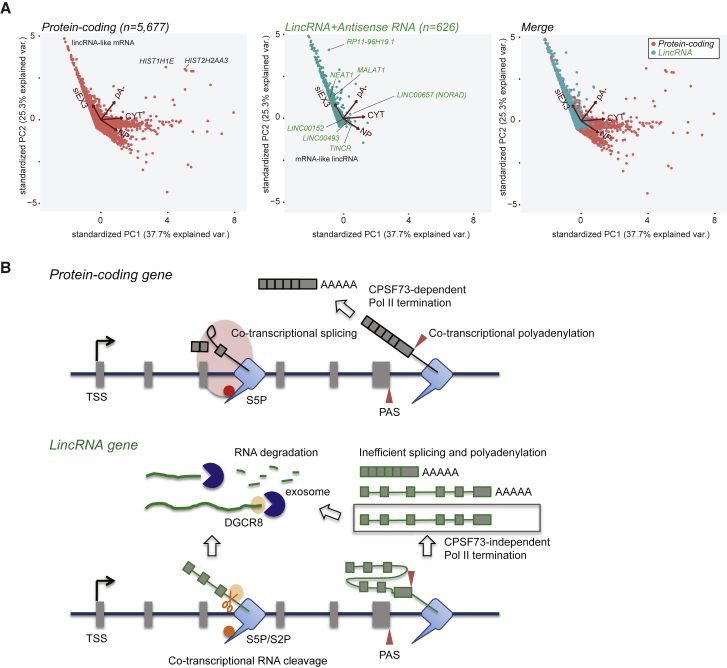
Protein Coding versus lincRNA Defining Features: PCA and Model (A) Principal-component analysis applied to protein-coding and lincRNA TUs shown separately and merged. Vectors indicating key parameters compared are shown by arrows: these are exosome sensitivity, pA−/pA+ levels, cytoplasmic/chromatin, and nucleoplasmic/chromatin levels. Some key lincRNAs are identified as well as some protein-coding transcript outliers. The graph has been cropped for better visualization, but PC1 and PC2 values of all data points are available in Table S2. (B) Model for protein-coding versus lincRNA co-transcriptional processing. Protein-coding genes are transcribed by Pol II with spliceosome (pink oblong) associated with CTD S5P (red dot). mRNA 3′ ends are generated co-transcriptionally by CPSF73 as part of CPA complex, which in turn promotes Pol II termination. lincRNA genes are weakly spliced and polyadenylated, resulting in CPSF73-independent termination and DGCR8-stimulated exosome degradation with co-transcriptional cleavage (scissors) associated with CTD S2P and S5P (orange dot) and exosome-mediated degradation on chromatin. See also Figure S7.

We employed principal-component analysis (PCA) to compare protein-coding versus lincRNA TUs based on multiple parameters. Because our restricted lincRNA set displays very similar profiles to the larger antisense lncRNA set (Figure 4F), these were combined for PCA. The effects of exosome knockdown on levels of nuclear RNA, nuclear-to-chromatin-associated RNA ratio, cytoplasmic-to-chromatin-associated RNA ratio, and the pA− to pA+ RNA ratio were collapsed into a two-dimensional representation in the principal components PC1 and PC2. The vectors depicted by arrows show the projection of the original four descriptors onto the PC1 and PC2 planes (Figure 7A). The main descriptor of lincRNA TUs is their upregulation upon exosome knockdown and their general lack of polyA. In contrast, the most distinguishing feature for protein-coding TUs is their stability within the nucleoplasm and cytoplasm. We note that a few lincRNAs behave in a similar manner to protein-coding TUs and are therefore potentially functional. Two clear examples are lincRNA *LINC00493* and *TINCR*, which are spliced, polyadenylated, and show an accumulation of nucleoplasm-spliced reads that lack exosome sensitivity (Figure S7A). Further examples of such potentially functional lincRNAs are listed (Table S2). We also analyzed protein-coding TUs, which have similar values in PC1 and PC2 to bulk lincRNA. Remarkably, the majority of these transcripts originate from an upstream promoter with respect to the main gene TSS (defined by higher chromatin-seq reads) and show significantly higher exosome sensitivity than transcripts from the main TSS (Figure S7B). In many cases, they derive from antisense transcripts (PROMPTs) emanating from an adjacent divergent protein-coding gene that will then read into the open reading frame (ORF) of the downstream gene (Table S2).

The full list of principal component (PC) values and the identified lincRNA-like protein-coding genes and protein-coding-like lincRNAs can be found in Table S2. Finally, it should be noted that PCA of lincRNAs derived from NONCODE without the elimination of overlapping TUs fails to show significant pattern differences with protein-coding genes (Figure S7C). Most of these lincRNAs behave similarly to protein-coding genes because they overlap with protein-coding genes or fall within their extended transcription termination regions. This emphasizes the importance of defining separate TUs to avoid lincRNA misidentification. Overall, we demonstrate that lincRNAs behave as a separate class of transcripts to protein-coding genes. They are co-transcriptionally cleaved by a Pol-II-associated endonuclease complex, which may in turn act to promote premature termination across lincRNA TUs (marked by T4P-specific mNET-seq profiles). Coupled to this, DGCR8 recognizes these 3′ ends and recruits the nuclear exosome to fully degrade these short-lived lincRNAs (Figure 7B).

## Discussion

We have analyzed HeLa cell nascent transcription using mNET-seq methodology (Nojima et al., 2015, Nojima et al., 2016), employing a full set of CTD phosphorylation-specific antibodies (Figure 1). Armed with this wide repertoire of CTD-specific nascent transcript profiles, we have been able to scrutinize potential differences between protein-coding and lincRNA genes. In general, protein-coding genes show higher selectivity for specific CTD modifications. Thus, unphosphorylated CTD (together with Y1P) is a hallmark of TSS-paused protein-coding gene transcripts whereas T4P CTD precisely defines their termination regions. S5P and S2P CTD profiles then match key co-transcriptional pre-mRNA processing states (splicing and 3′ end cleavage and polyadenylation). In contrast, lincRNA CTD profiles appear less selective with all the above-mentioned CTD tendencies of protein-coding genes diminished. Whereas Pol II pausing at the TSS and TES of protein-coding genes appears to be a tightly regulated process, this is generally absent for lincRNA genes. Similarly, the dominant RNA-processing reactions, co-transcriptional splicing, and 3′ cleavage and polyadenylation are associated with precise CTD marks S5P and S2P. Again, lincRNAs, which are largely unspliced (Figure 2) and generally not 3′ end processed (Figure 3), lack these dominant phospho-CTD features. Because this RNA processing is required to generate translatable mRNAs, it appears logical that noncoding lincRNAs lack the transcriptional CTD code that enhances these processes.

We observe less Pol II pausing over the TES region of lincRNA genes, compared to protein-coding genes (Figure S1B, bottom). Protein-coding gene TES pausing depends on CPA factors, such as CPSF73 and CstF64/64 tau using unph, S2P, and S5P Pol II CTD antibodies (Nojima et al., 2015). Here, we show that the mNET-seq/T4P profile gives the largest Pol II read accumulation in the TES region of protein-coding genes. Whereas this pausing effect at the TES is decreased by depletion of CPSF73 protein (Figure 3C, left), the profile switches to other T4P CTD peaks further downstream (Figures 3A and S3B). We hypothesize that the observed downstream CPA-independent termination is a failsafe mechanism. Possibly, additional terminators beyond CPA-dependent mechanisms are generally present to restrict transcriptional interference caused by uncontrolled transcriptional readthrough (Greger and Proudfoot, 1998, Rutkowski et al., 2015). Interestingly, mNET-seq/T4P peaks at lincRNA TES are in general CPSF73 independent (Figures 3B, 3D, and S3C). Some lincRNAs retain CPA-independent termination, even though they lack CPA-dependent mechanisms. Consistent with this result, we also confirm lincRNAs are in general inefficiently 3′ end polyadenylated (Figure 3F). We note that mNET-seq/T4P signals in the lincRNA gene body are often decreased by CPSF73 knockdown (Figure S3C). This suggests that premature termination of lincRNAs may still be regulated by CPA factors.

Our analysis of HeLa cell lincRNAs by subcellular RNA-seq analysis reveals a clear pathway to their rapid degradation (Figure 7). First, we show that lincRNAs are mainly restricted to the nuclear chromatin fraction, as observed for eRNAs, PROMPTs, and antisense RNAs. We also demonstrate that chromatin-restricted lincRNAs are degraded by the nuclear exosome as soon as they are made (Figures 4E, 4F, S4B, and S4C). However, to be substrates for exosome-associated 3′ exonuclease, lincRNAs must first be cleaved by endonucleases to generate accessible 3′ ends. Our mNET-seq analysis of lincRNAs using Empigen treatment indicates the presence of a separable endonuclease complex associated with Pol II. Thus, Empigen treatment removes multiple cleavage sites across lincRNAs, which are detectable as peaks in the mNET-seq analysis. These RNA 3′ ends do not derive from splicing because their appearance is insensitive to the splicing inhibitor Pla-B.

We examined the possibility that lincRNA endonucleolytic cleavage could be generally mediated by the microprocessor. Components of microprocessor, Drosha, and DGCR8 proteins cleave pre-miRNA structures co-transcriptionally (Morlando et al., 2008, Nojima et al., 2015). We therefore suspected that lincRNAs might possess multiple pre-miRNA-like secondary structures and so be cleaved by the microprocessor. Depletion of DGCR8 (which causes inactivation of the microprocessor) followed by mNET-seq analysis removed mNET-seq peaks corresponding to authentic pre-miRNAs (Figure 6B). However, unexpectedly, Empigen-sensitive cleavage sites on lincRNAs were generally increased by DGCR8 knockdown (Figures 6 and S5B). Because DGCR8 is both associated with elongating Pol II and with RNA exosome components, it is likely to enhance exosome activity. It is, however, also possible that DGCR8 plays a regulatory role in the recruitment or activity of the presumptive lincRNA endonuclease. Overall, we propose a model for lincRNA degradation in which these weakly spliced and polyadenylated transcripts are largely degraded post-transcriptionally by DGCR8-mediated recruitment of the nuclear exosome (Figure 7B). Another feature of lincRNA transcription is that many transcripts prematurely terminate well before reaching the distal TES.

We ended our bioinformatics comparison of lincRNA TUs versus protein-coding TUs by subjecting them to PCA (Figure 7A). Remarkably, lincRNAs gave a characteristic profile showing high exosome sensitivity. However, a few lincRNAs display more protein-coding-like properties (Figure S7A; Table S2) and so may represent transcripts with specific functions. Notably, protein-coding TUs gave a mainly non-overlapping PC profile with lincRNA TUs. Those that did significantly match the lincRNA PC profile correspond to transcripts derived from upstream start sites and often come from divergent gene PROMPTs. These can therefore be viewed as lincRNA TUs. Overall, our bioinformatics comparison of lincRNA versus protein-coding TUs underlies substantial differences between these two transcript classes. In general, lincRNAs appear unlikely to possess sequence-specific functions. Possibly, the act of transcription rather than the nature of the transcript underlies their biological purpose. However, it remains an attractive possibility that tissue-specific RNA-binding proteins (possibly absent in HeLa cells) may selectively restrict lincRNA turnover and so allow their sufficient accumulation to promote functional roles at least for some of these RNAs.

## Experimental Procedures

### mNET-Seq and Fractionated RNA-Seq

Detailed protocols for mNET-seq, ChrRNA-seq, and NpRNA-seq were previously described (Nojima et al., 2015, Nojima et al., 2016). For mNET-seq/total, unph, S2P, and S5P, published data were used (Nojima et al., 2015).

### Transcription Unit Annotation

Hg19/GRCh37 was used as a reference genome. TUs were extracted based on ENSEMBL (GRCh37.75; Flicek et al., 2014), NONCODE v4 (Xie et al., 2014), and UCSC tRNA (Lowe and Eddy, 1997). PROMPTs were extracted based on published data (Ntini et al., 2013), and ubiquitously expressed eRNAs were taken from PrESSTo (FANTOM 5 project; Andersson et al., 2014). Overlapping, expressed TUs and exons were reduced to the most upstream and downstream boundaries. Some overlapping TUs with different biotypes were excluded from further analysis. Defined TUs were categorized by biotype (Tables S1 and S2).

### Data Processing

RNA-sequencing reads were trimmed by Cutadapt 1.8.3 and then mapped to the human hg19 reference sequence with Tophat 2.0.13. All sequencing data were processed to only include properly paired, properly mapped reads with SAMtools 1.2. mNET-seq profiles were created by only using the most 3′ nucleotide of the second sequencing read. Data were visualized with Bedtools 2.23.0 and scaled to each library size (genomeCoverageBed).

### Bioinformatic Analysis

Heatmaps were created using the MATLAB R2015b image function. All other graphs were created using ggplot2 in R. p values are computed via a Wilcoxon test in R or a Fisher exact test in MATLAB (Figure 2D). PCA is based on the R prcomp function and visualized with ggbiplot.

## Author Contributions

M.S., with advice from T.G., performed all bioinformatics analyses. T.N. performed all molecular biology and transcriptomic experiments with help from A.D. on the microprocessor. N.J.P. and T.N. designed the project and wrote the paper with help from M.S. and M.C.-F.

## Accession Numbers

The accession number for the sequencing data reported in this paper is GEO: GSE81662.

## Supplemental Information

Document S1. Supplemental Experimental Procedures, Figures S1–S7, and Table S1

Table S2. Supporting Gene Lists Used for Genomic Analyses and PCA, Related to Experimental Procedures Dataset 1 is a list of all genomic regions used for the analyses in Figures 1-4. Many overlapping genes and genes with low expression in HeLa cells were excluded (Supplemental Experimental Procedures). Dataset 2 is a list of all lincRNA genes used for PCA from Dataset 1. Descriptors were computed based on RNA-Seq data from Mayer et al. (2015) (GEO:GSE61332). LincRNA genes were excluded if the chromatin signal from Mayer et al. (2015) was 0. Data are sorted in decreasing order of PC1. Dataset 3 is as Dataset 2 but for antisense genes. Dataset 4 is a list of all lincRNA genes used for PCA ENSEMBL and NONCODE. Less stringent criteria for allowing overlapping transcription units were used. Descriptors, inclusion of lincRNA genes, and sorting are equivalent to Dataset 2. Dataset 5 is equivalent of Dataset 2 but for protein coding genes. Dataset 6 is equivalent of Dataset 4 but for protein coding genes. Dataset 7 is a list of coding genes from the PCA with multiple TSSs, which behave similar to lincRNA (PC1 < 0, PC2 > 1). TSS1 refers to the upstream TSS used in the PCA. TSS2 refers to a downstream TSS, which was not used in the analysis. Green highlights the genes where the downstream TSS is mainly used according to higher Seq signal on the chromatin. Purple highlights the genes where the upstream TSS may be mainly used according to chromatin Seq signal. These data are based on our RNA Seq data from chromatin (Chr), nucleoplasm (NP) siLuc, and NP siEX3. Dataset 8 is equivalent to Dataset 7, but for coding genes with one annotated TSS. Comments indicate which genes may be overlapping a PROMPT, giving rise to the observed effect. Comments also indicate where there may be a misannotation in the database or where UCSC and ENSEMBL entries do not match. Dataset 9 is equivalent to Dataset 7 but with inclusion of lincRNA from ENSEMBL and NONCODE (corresponding to the lincRNA from Dataset 4). Dataset 10 is equivalent to Dataset 8 but with inclusion of lincRNA from ENSEMBL and NONCODE (corresponding to the lincRNA from Dataset 4).

Document S2. Article plus Supplemental Information
