# Supplementary material for: Distinctive Patterns of Transcription and RNA Processing for Human lincRNAs
Source: Mol Cell. 2017 Jan 5;65(1):25–38. doi: 10.1016/j.molcel.2016.11.029 (PMC5222723; doi:10.1016/j.molcel.2016.11.029)
Supplement: Document S1. Supplemental Experimental Procedures, Figures S1–S7, and Table S1 [file mmc1.pdf]

Molecular Cell, Volume *65*

Supplemental Information

Distinctive Patterns of Transcription and RNA Processing for Human lincRNAs

Margarita Schlackow, Takayuki Nojima, Tomas Gomes, Ashish Dhir, Maria Carmo-Fonseca, and Nick J. Proudfoot

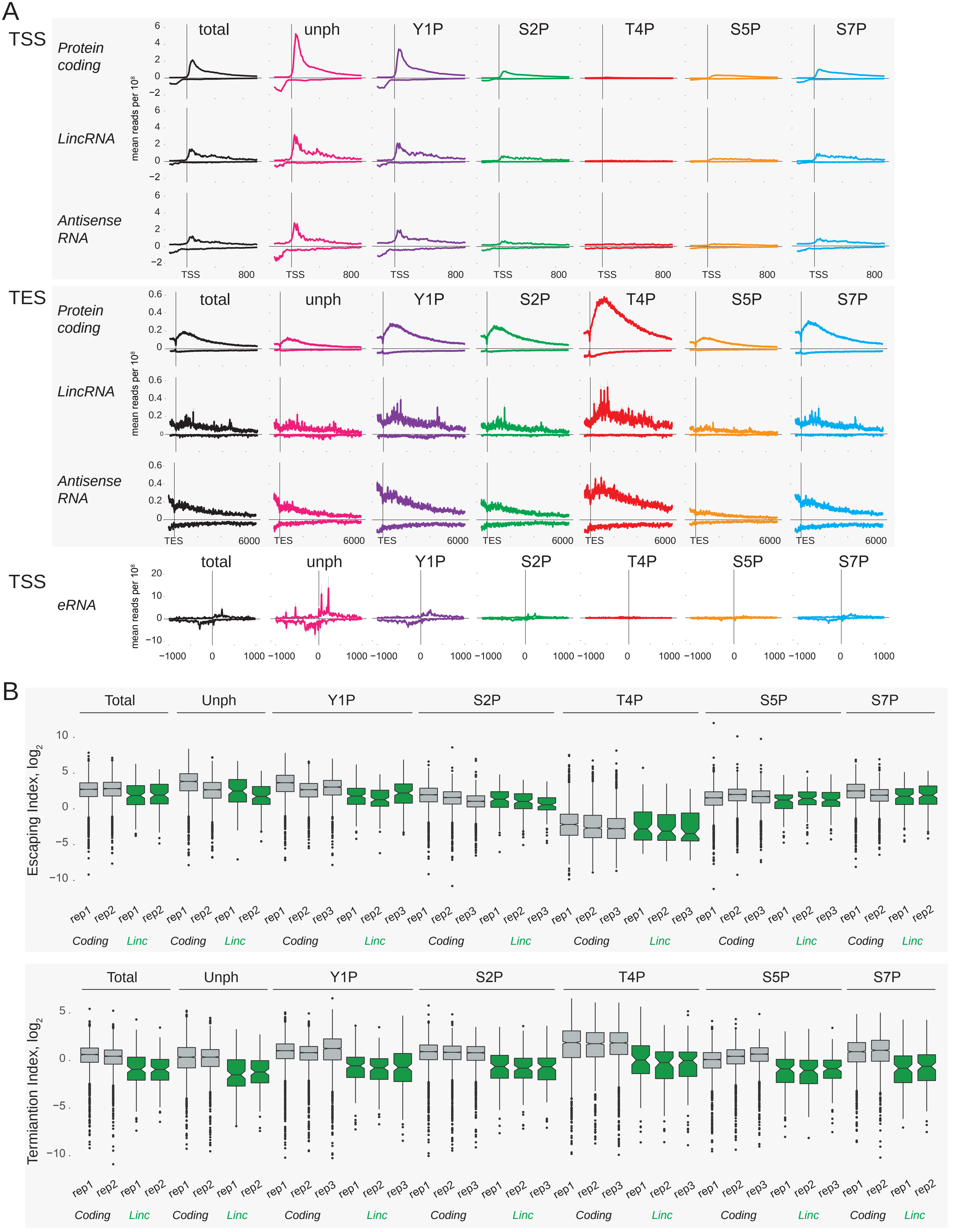
A
TSS
Protein coding
LincRNA
Antisense RNA
mean reads per 10⁸
total
unph
Y1P
S2P
T4P
S5P
S7P
TSS
800
TES
6000
eRNA
-1000
0
1000
B
Total
Unph
Escaping Index, log2
Termiantion Index, log2
rep1
rep2
rep3
Coding
Linc

Figure S1

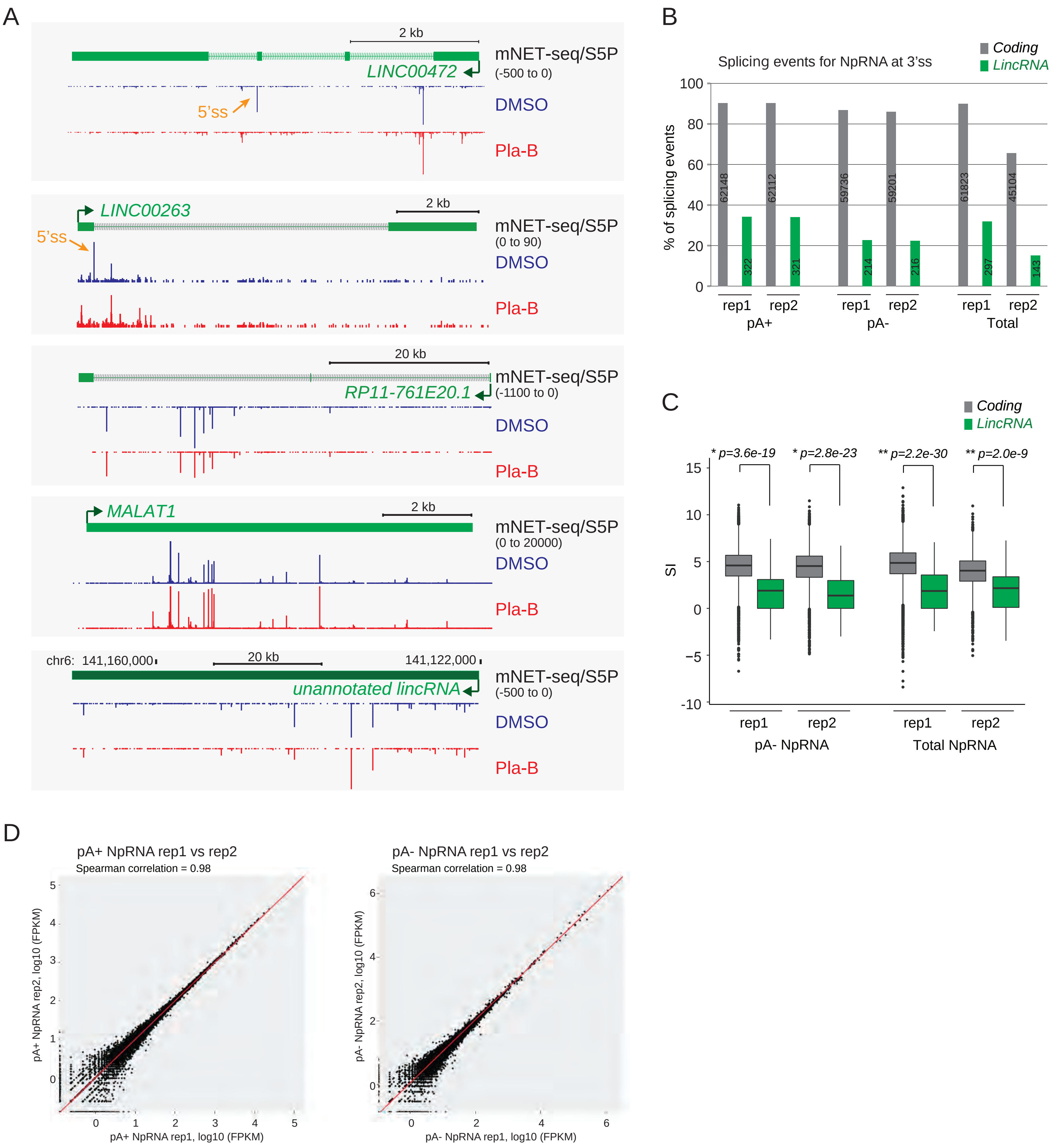

A
2 kb
LINC00472
mNET-seq/S5P
(-500 to 0)
5'ss
DMSO
Pla-B
LINC00263
2 kb
5'ss
mNET-seq/S5P
(0 to 90)
DMSO
Pla-B
20 kb
RP11-761E20.1
mNET-seq/S5P
(-1100 to 0)
DMSO
Pla-B
MALAT1
2 kb
mNET-seq/S5P
(0 to 20000)
DMSO
Pla-B
chr6: 141,160,000
20 kb
141,122,000
unannotated lincRNA
mNET-seq/S5P
(-500 to 0)
DMSO
Pla-B
B
Splicing events for NpRNA at 3'ss
Coding
LincRNA
% of splicing events
62148
322
62112
321
59736
214
59201
216
61823
297
45104
143
rep1
rep2
pA+
pA-
Total
C
Coding
LincRNA
* p=3.6e-19
* p=2.8e-23
** p=2.2e-30
** p=2.0e-9
SI
pA- NpRNA
Total NpRNA
D
pA+ NpRNA rep1 vs rep2
Spearman correlation = 0.98
pA+ NpRNA rep2, log10 (FPKM)
pA+ NpRNA rep1, log10 (FPKM)
pA- NpRNA rep1 vs rep2
Spearman correlation = 0.98
pA- NpRNA rep2, log10 (FPKM)
pA- NpRNA rep1, log10 (FPKM)

Figure S2

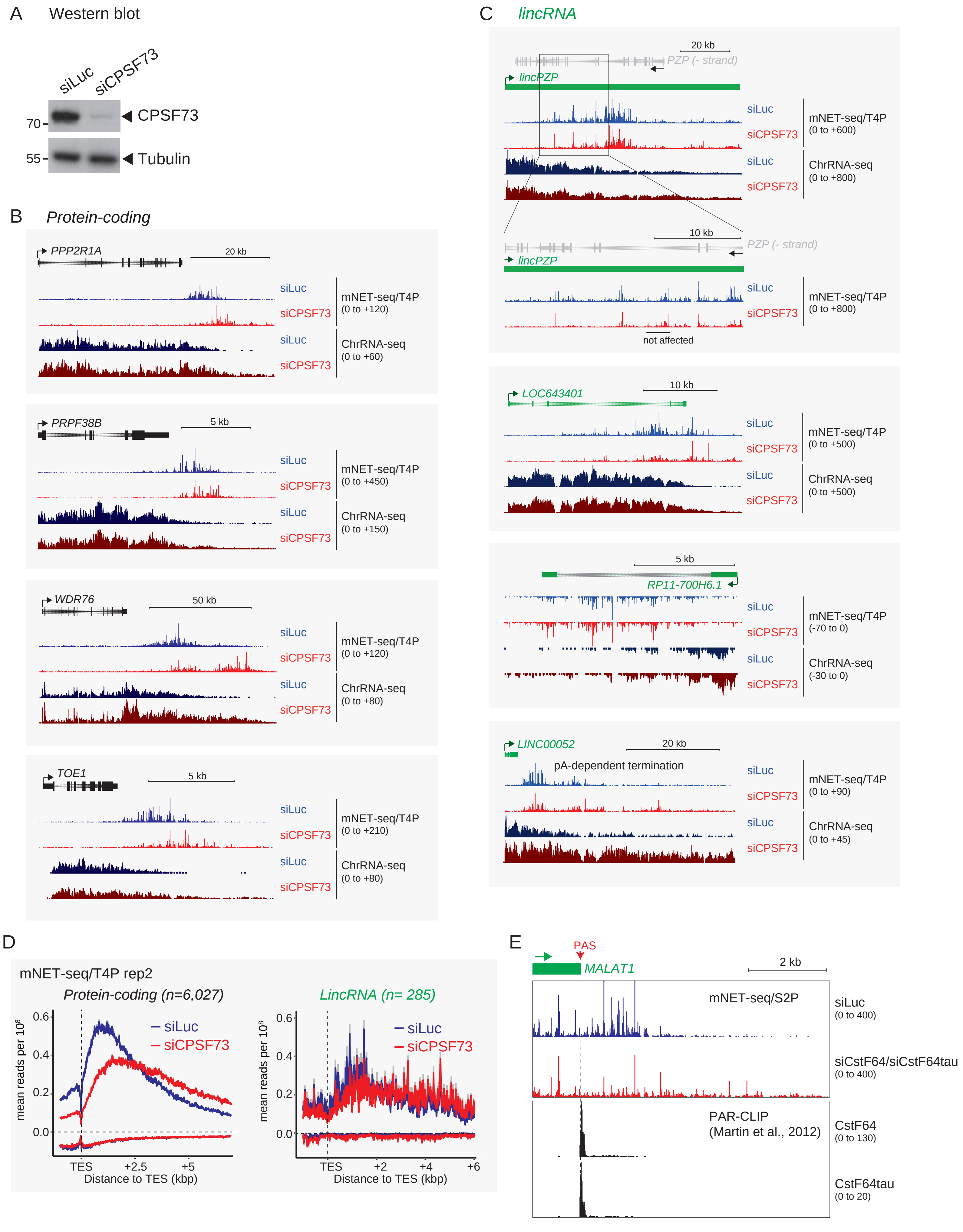
A
Western blot
siLuc
siCPSF73
70
55
CPSF73
Tubulin
B
Protein-coding
PPP2R1A
20 kb
siLuc
siCPSF73
mNET-seq/T4P
(0 to +120)
ChrRNA-seq
(0 to +60)
PRPF38B
5 kb
mNET-seq/T4P
(0 to +450)
ChrRNA-seq
(0 to +150)
WDR76
50 kb
mNET-seq/T4P
(0 to +120)
ChrRNA-seq
(0 to +80)
TOE1
5 kb
mNET-seq/T4P
(0 to +210)
ChrRNA-seq
(0 to +80)
C
lincRNA
20 kb
PZP (- strand)
lincPZP
mNET-seq/T4P
(0 to +600)
ChrRNA-seq
(0 to +800)
10 kb
PZP (- strand)
lincPZP
mNET-seq/T4P
(0 to +800)
not affected
LOC643401
10 kb
mNET-seq/T4P
(0 to +500)
ChrRNA-seq
(0 to +500)
5 kb
RP11-700H6.1
mNET-seq/T4P
(-70 to 0)
ChrRNA-seq
(-30 to 0)
LINC00052
20 kb
pA-dependent termination
mNET-seq/T4P
(0 to +90)
ChrRNA-seq
(0 to +45)
D
mNET-seq/T4P rep2
Protein-coding (n=6,027)
LincRNA (n= 285)
mean reads per 10⁸
0.6
0.4
0.2
0.0
TES
+2.5
+5
+2
+4
+6
Distance to TES (kbp)
E
PAS
MALAT1
2 kb
mNET-seq/S2P
siLuc
(0 to 400)
siCstF64/siCstF64tau
(0 to 400)
PAR-CLIP
(Martin et al., 2012)
CstF64
(0 to 130)
CstF64tau
(0 to 20)

Figure S3

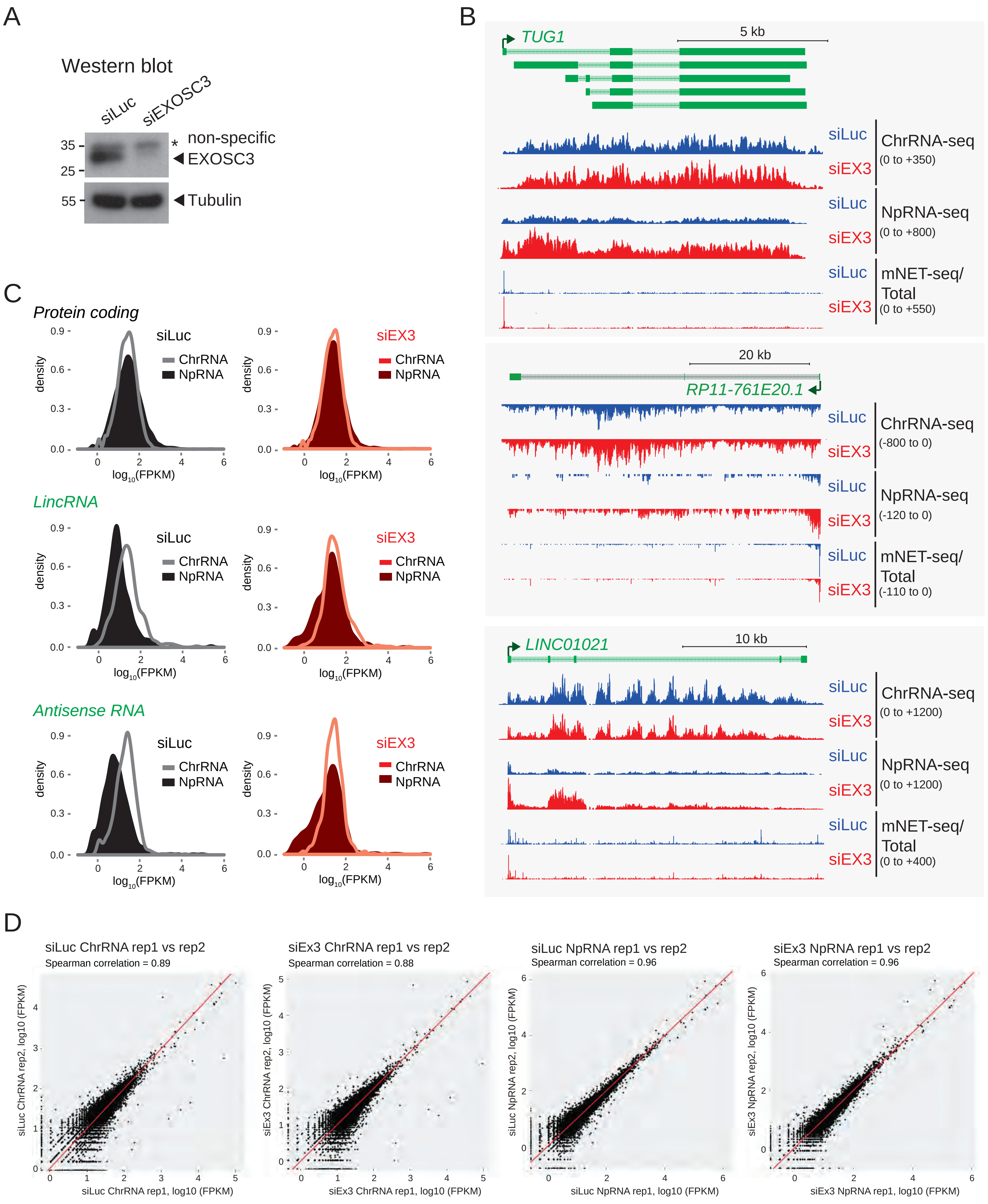
A
Western blot
siLuc
siEXOSC3
35
25
55
* non-specific
EXOSC3
Tubulin
B
TUG1
5 kb
siLuc
siEX3
ChrRNA-seq
(0 to +350)
NpRNA-seq
(0 to +800)
mNET-seq/
Total
(0 to +550)
20 kb
RP11-761E20.1
ChrRNA-seq
(-800 to 0)
NpRNA-seq
(-120 to 0)
mNET-seq/
Total
(-110 to 0)
LINC01021
10 kb
ChrRNA-seq
(0 to +1200)
NpRNA-seq
(0 to +1200)
mNET-seq/
Total
(0 to +400)
C
Protein coding
LincRNA
Antisense RNA
siLuc
siEX3
ChrRNA
NpRNA
density
log10(FPKM)
D
siLuc ChrRNA rep1 vs rep2
Spearman correlation = 0.89
siEx3 ChrRNA rep1 vs rep2
Spearman correlation = 0.88
siLuc NpRNA rep1 vs rep2
Spearman correlation = 0.96
siEx3 NpRNA rep1 vs rep2
Spearman correlation = 0.96
siLuc ChrRNA rep1, log10 (FPKM)
siLuc ChrRNA rep2, log10 (FPKM)
siEx3 ChrRNA rep1, log10 (FPKM)
siEx3 ChrRNA rep2, log10 (FPKM)
siLuc NpRNA rep1, log10 (FPKM)
siLuc NpRNA rep2, log10 (FPKM)
siEx3 NpRNA rep1, log10 (FPKM)
siEx3 NpRNA rep2, log10 (FPKM)

Figure S4

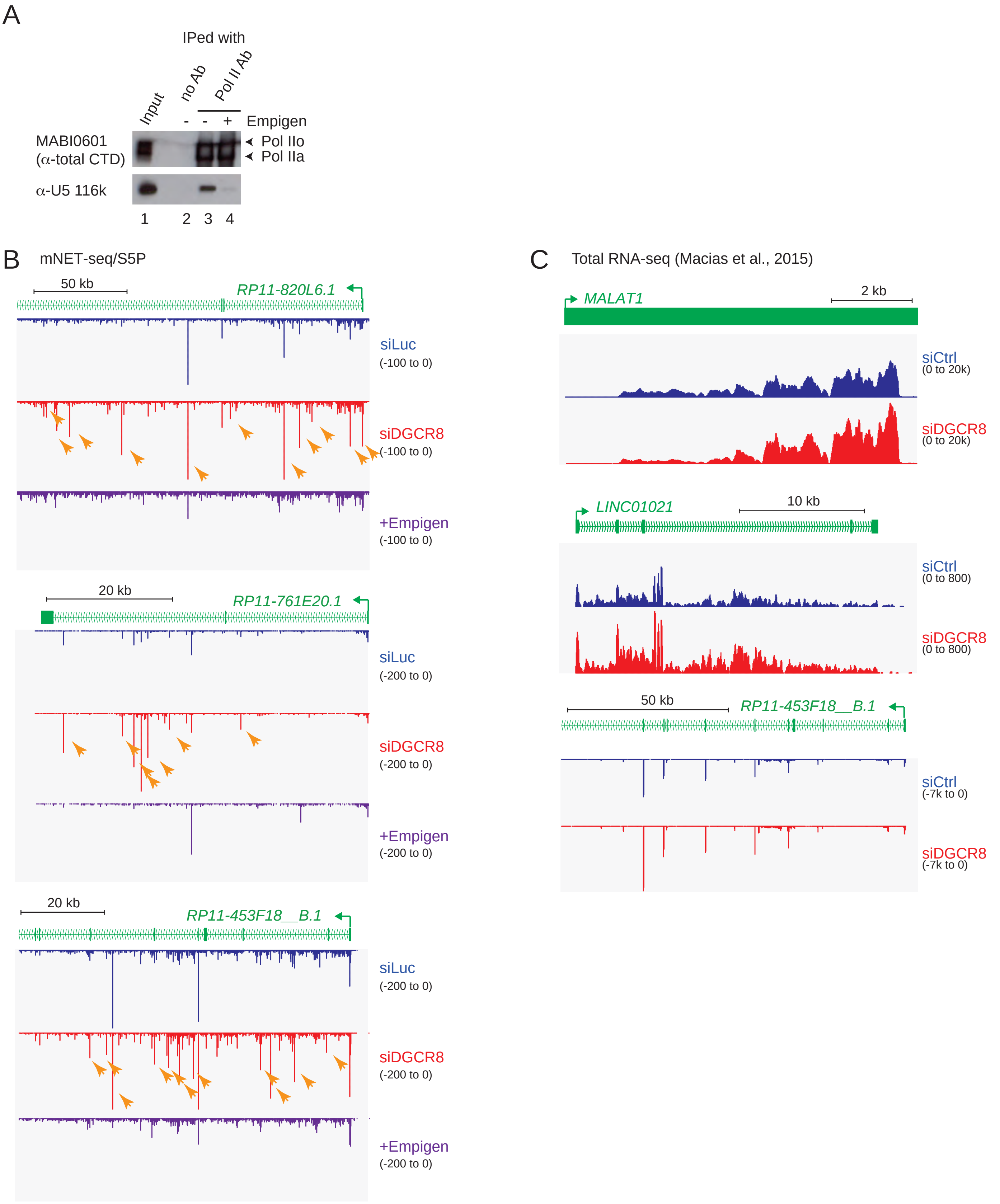

A
IPed with
Input
no Ab
Pol II Ab
- - + Empigen
MABI0601
(α-total CTD)
Pol IIo
Pol IIa
α-U5 116k
1 2 3 4
B
mNET-seq/S5P
50 kb
RP11-820L6.1
siLuc
(-100 to 0)
siDGCR8
(-100 to 0)
+Empigen
(-100 to 0)
20 kb
RP11-761E20.1
siLuc
(-200 to 0)
siDGCR8
(-200 to 0)
+Empigen
(-200 to 0)
20 kb
RP11-453F18__B.1
siLuc
(-200 to 0)
siDGCR8
(-200 to 0)
+Empigen
(-200 to 0)
C
Total RNA-seq (Macias et al., 2015)
MALAT1
2 kb
siCtrl
(0 to 20k)
siDGCR8
(0 to 20k)
LINC01021
10 kb
siCtrl
(0 to 800)
siDGCR8
(0 to 800)
50 kb
RP11-453F18__B.1
siCtrl
(-7k to 0)
siDGCR8
(-7k to 0)

Figure S5

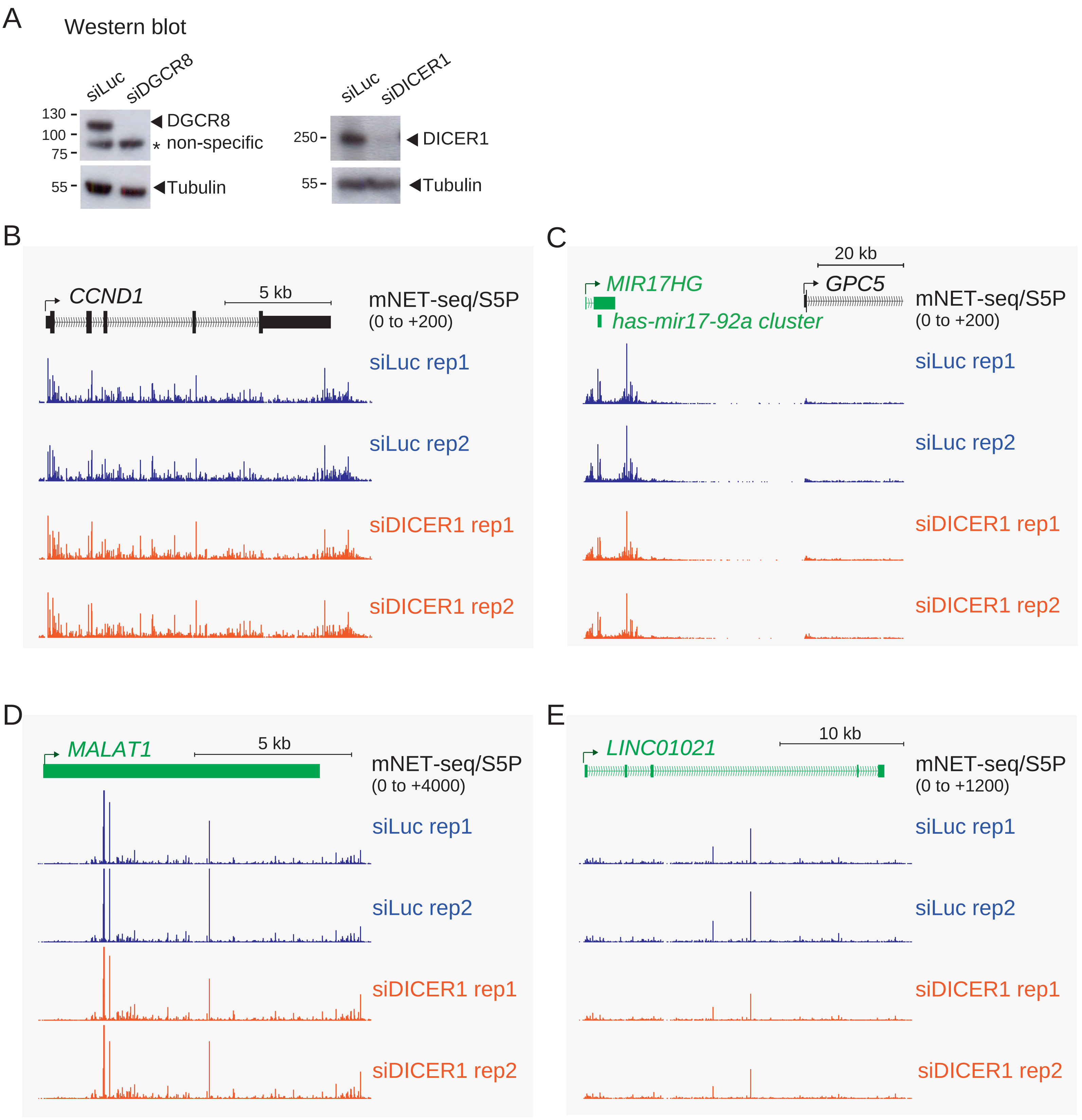
A
Western blot
siLuc
siDGCR8
siLuc
siDICER1
130
100
75
55
DGCR8
* non-specific
Tubulin
250
55
DICER1
Tubulin
B
CCND1
5 kb
mNET-seq/S5P
(0 to +200)
siLuc rep1
siLuc rep2
siDICER1 rep1
siDICER1 rep2
C
20 kb
MIR17HG
GPC5
has-mir17-92a cluster
mNET-seq/S5P
(0 to +200)
siLuc rep1
siLuc rep2
siDICER1 rep1
siDICER1 rep2
D
MALAT1
5 kb
mNET-seq/S5P
(0 to +4000)
siLuc rep1
siLuc rep2
siDICER1 rep1
siDICER1 rep2
E
LINC01021
10 kb
mNET-seq/S5P
(0 to +1200)
siLuc rep1
siLuc rep2
siDICER1 rep1
siDICER1 rep2

Figure S6

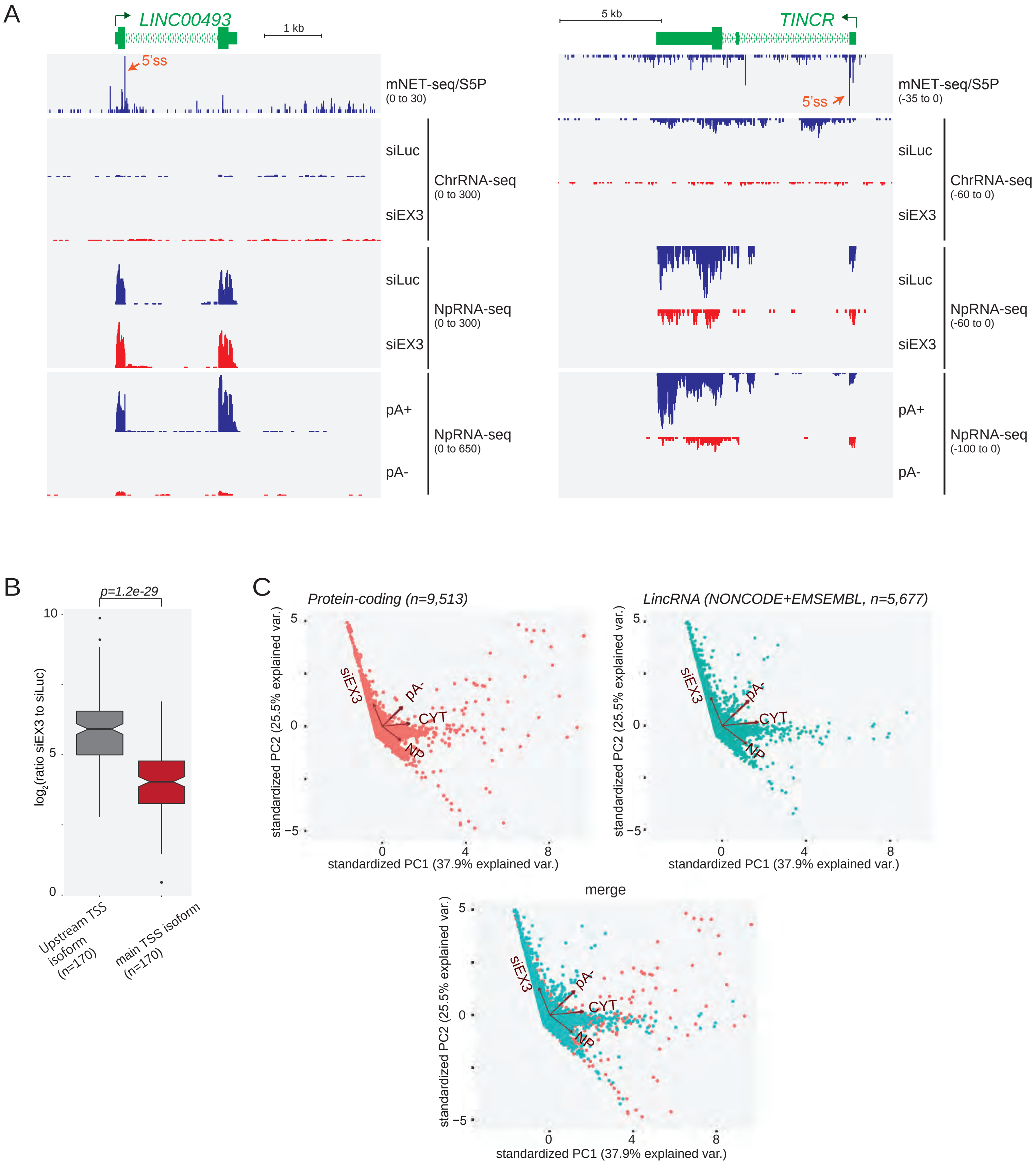
A
LINC00493
1 kb
5'ss
mNET-seq/S5P
(0 to 30)
siLuc
ChrRNA-seq
(0 to 300)
siEX3
siLuc
NpRNA-seq
(0 to 300)
siEX3
pA+
NpRNA-seq
(0 to 650)
pA-
5 kb
TINCR
5'ss
mNET-seq/S5P
(-35 to 0)
siLuc
ChrRNA-seq
(-60 to 0)
siEX3
siLuc
NpRNA-seq
(-60 to 0)
siEX3
pA+
NpRNA-seq
(-100 to 0)
pA-
B
p=1.2e-29
log2(ratio siEX3 to siLuc)
Upstream TSS isoform (n=170)
main TSS isoform (n=170)
C
Protein-coding (n=9,513)
LincRNA (NONCODE+EMSEMBL, n=5,677)
merge
siEX3
pA-
CYT
NP
standardized PC1 (37.9% explained var.)
standardized PC2 (25.5% explained var.)

Figure S7

**ENSEMBL Gene Biotype**	**Simplified category**
IG_C_gene	coding
IG_D_gene	coding
IG_J_gene	coding
IG_LV_gene	coding
IG_V_gene	coding
TR_C_gene	coding
TR_J_gene	coding
TR_V_gene	coding
TR_D_gene	coding
IG_C_pseudogene	pseudogene
IG_J_pseudogene	pseudogene
IG_V_pseudogene	pseudogene
TR_V_pseudogene	pseudogene
TR_J_pseudogene	pseudogene
Mt_rRNA	ncRNA
Mt_tRNA	ncRNA
miRNA	ncRNA
misc_RNA	ncRNA
rRNA	ncRNA
snRNA	ncRNA
snoRNA	ncRNA
ribozyme	ncRNA
sRNA	ncRNA
scaRNA	ncRNA
Mt_tRNA_pseudogene	pseudogene
tRNA_pseudogene	pseudogene
snoRNA_pseudogene	pseudogene
snRNA_pseudogene	pseudogene
scRNA_pseudogene	pseudogene
rRNA_pseudogene	pseudogene
misc_RNA_pseudogene	pseudogene
miRNA_pseudogene	pseudogene
TEC	predicted
nonsense_mediated_decay	discarded
non_stop_decay	pseudogene
retained_intron	discarded
protein_coding	coding
processed_transcript	lincRNA
non_coding	ncRNA
ambiguous_orf	predicted
sense_intronic	discarded
sense_overlapping	ncRNA
antisense	antisense
known_ncrna	ncRNA

pseudogene	pseudogene
processed_pseudogene	pseudogene
polymorphic_pseudogene	pseudogene
retrotransposed	pseudogene
transcribed_processed_pseudogene	pseudogene
transcribed_unprocessed_pseudogene	pseudogene
transcribed_unitary_pseudogene	pseudogene
translated_unprocessed_pseudogene	pseudogene
unitary_pseudogene	pseudogene
unprocessed_pseudogene	pseudogene
artifact	discarded
lincRNA	lincRNA
macro_lncRNA	lncrna
LRG_gene	discarded
3prime_overlapping_ncrna	ncRNA
disrupted_domain	discarded
vaultRNA	ncRNA

Table S1

SUPPLEMENTARY INFORMATION

SUPPLEMENTARY FIGURE AND TABLE LEGENDS

Figure S1 (related to Figure 1)

(A) Meta-analysis of different transcript categories (as indicated) using mNET-seq analysis with indicated Pol II antibodies centered over either TSS or TES except for eRNA category which is centered over sense and antisense TSS.

(B) Quantitation (box plots) of TSS Escaping index and TES Termination index for protein coding versus lincRNA meta-analysis. Replicated data is presented in all cases

Figure S2 (related to Figure 2)

(A) mNET-seq/S5P profiles for indicated lincRNAs with transcript read profiles aligned to above gene maps. HeLa cells were treated with Pla-B (in DMSO) or mock treated with DMSO. Arrow denotes promoter direction. Yellow arrows denote high 5'ss PLa-B sensitive mNET-seq signals.

(B) Tabulation of splicing event % (total numbers indicated in bars) for coding or lincRNA in total or pA+/- nuclear RNA. Duplicate data is presented.

(C) Splicing index derived from pA- or total NpRNA-seq. Duplicate data is presented.

(D) Reproducibility is tested via scatter plots for FPKM values (first 500 nt) in pA+ and pA- NpRNA-seq duplicates. Spearman coefficient confirms a high positive correlation.

Figure S3 (related to Figure 3)

(A) Western blot showing degree of CPSF73 depletion following siCPSF73 but not siLuc treatments of Hela cells. Blots with anti CPSF73 and control anti tubulin are shown.

(B) mNET-seq/T4P versus chromatin RNA-seq profiles are shown for four protein-coding genes as indicated from chromatin extracted siLuc or siCPSF73 treated HeLa cells.

(C) As for (B) but for 4 lincRNA TUs. For *lincPZP*, antisense the protein coding gene PZP (not expressed in HeLa cells) a blow up of boxed region is also shown.

(D) Meta-analysis of termination region as shown in Figure 3C but using duplicate data.

(E) mNET-seq/S2P and PAR-CLIP data for *MALAT1* 3' end region.

Figure S4 (related to Figure 4)

(A) Western blot showing degree of exosome component EXOSC3 depletion following siEXOSC3 but not siLuc treatments of Hela cells. Blots with anti EXOSC3 and control anti tubulin are shown. * denotes nonspecific band.

(B) Profile comparison between ChrRNA-seq, NpRNA-seq and mNET-seq profiles with and without EXOSC3 depletion for three lincRNA TUs as indicated.

(C) Density plots of FPKM levels for indicated RNA types with or without EXOSC3 depletion comparing protein-coding, lincRNA and antisense RNA TUs.

(D) Scatter plots of FPKM values (first 500 nt) showing high reproducibility (Spearman correlation coefficient indicated) of ChrRNA-seq and NpRNA-seq with mock or siEXOSC3 depleted HeLa cells.

Figure S5 (related to Figure 5 and 6)

(A) Co-immunoprecipitation of spliceosomal U5 116k protein with Pol II blocked by empigen treatment.

(B) mNET-seq/S5P profiles for indicated lincRNA TUs from HeLa cells treated with siLuc (control), siDGCR8 (see Figure S6A) or micrococcal nuclease digested chromatin pretreated with empigen (see Supplementary Methods). Positions of dominant multiple DGCR8

sensitive peaks are indicated by yellow arrows.

(C) Total RNA-seq profiles for indicated lincRNA with or without DGCR8 depletion by siRNA treatment.

Figure S6 (related to Figure 6)

(A) Western blots showing degree of DGCR8 and Dicer depletion following siDGCR8 or siDICER1 but not siLuc treatments of Hela cells. Blots with anti DGCR8 and anti DICER1 versus control anti tubulin are shown. * denotes nonspecific band.

(B-E) mNET-seq/S5P profiles for indicated lincRNA TUs with or without DICER1 depletion. For *MIR17HG* (C) downstream tandem protein-coding gene *GPC5* is also presented. Note that all duplicated profiles are shown.

Figure S7 (related to Figure 7)

(A) Specific examples of protein coding-like lincRNA showing profiles of mNET-seq/S5P, ChrRNA-seq and NpRNA-seq with or without EXOSC3 depletion and NpRNA-seq pA- and pA+ selected. Position of 5’SS mNET-seq peak is indicated by arrow.

(B) Protein coding genes, which appear EXOC3 sensitive show weakened EXOC3 sensitivity if a more dominant, further downstream TSS is considered.

(C) Principal component analysis applied to protein coding and lincRNA TUs shown separately and merged as in Figure 7. However in this case the NONCODE data base (Xie et al., 2014) is used as the main source of lincRNA and protein-coding TUs.

Table S1 (related to Experimental Procedures)

Simplified categories used instead of the original ENSEMBL gene biotype to guide transcription unit annotation.

Table S2 (related to Experimental Procedures)

Dataset 1 is a list of all genomic regions used for the analyses in Figures 1-4. Many overlapping genes and genes with low expression in HeLa cells were excluded (Supplemental Experimental Procedures).

Dataset 2 is a list of all lincRNA genes used for PCA from Dataset 1. Descriptors were computed based on RNA-Seq data from Mayer et al. 2015 (GEO:GSE61332). LincRNA genes were excluded if the chromatin signal from Mayer et al. 2015 was 0. Data is sorted in decreasing order of PC1.

Dataset 3 is as Dataset 2 but of antisense genes.

Dataset 4 is a list of all lincRNA genes used for PCA ENSEMBL and NONCODE. Less stringent criteria for allowing overlapping transcription units were used. Descriptors, inclusion of lincRNA genes and sorting is equivalent to Dataset 2.

Dataset 5 is equivalent of Dataset 2 but of protein coding genes.

Dataset 6 is equivalent of Dataset 4 but of protein coding genes.

Dataset 7 is a list of coding genes from the PCA with multiple TSSs, which behave similar to lincRNA ($PC1<0$, $PC2>1$). TSS1 refers to the upstream TSS used in the PCA. TSS2 refers to a downstream TSS, which was not used in the analysis. Green highlights the genes where the downstream TSS is mainly used according to higher Seq signal on the chromatin. Purple highlights the genes where the upstream TSS may be mainly used according to chromatin Seq signal. This data is based on our RNA Seq data from chromatin (Chr), nucleoplasm (NP) siLuc and NP siEX3.

Dataset 8 is equivalent to Dataset 7, but of coding genes with one annotated TSS. Comments indicate which genes may be overlapping a PROMPT giving rise to the observed effect. Comments also indicate where there may be a misannotation in the database or where UCSC and ENSEMBL entries don't match.

Dataset 9 is equivalent to Dataset 7 but with inclusion of lincRNA from ENSEMBL and NONCODE (corresponding to the lincRNA from Dataset 4).

Dataset 10 is equivalent to Dataset 8 but with inclusion of lincRNA from ENSEMBL and NONCODE (corresponding to the lincRNA from Dataset 4)

Methods

siRNA transfection

SMARTpool siRNA against human CPSF73 (CPSF3), EXOSC3 and Dicer were purchased from Thermo scientific. DGCR8 siRNA is previously described (Dhir et al., 2015). These siRNA (final concentration 30 nM) were transfected into HeLa cells using Lipofectamine RNAiMAX reagent (Life technologies) according to the manual and incubated for between 60 and 72 hr.

Antibodies

Pol II antibodies CMA601, CMA602 and CMA603 were purchased from MBL international (Nojima et al., 2016). Pol II antibodies 4E12 (phospho Ser7) and 6D7 (phospho Thr4) were purchased from active motif. Pol II antibody 3D12 (phospho Tyr1) was purchased from Millipore. Pol II antibody 8WG16, Dicer and Drosha antibodies were purchased from Abcam. DGCR8 and Tubulin antibodies were purchased from Novus bio and Sigma, respectively. CPSF73, SNRP116 (U5 116k) and EXOC3 antibodies were purchased from Bethyl.

mNET-seq method

The detailed protocol was as previously described (Nojima et al., 2016).

Fractionated RNA-seq methods

ChrRNA-seq and NpRNA-seq method including the library preparation method were as previously described (Nojima et al., 2015).

pA+/- RNA selection

pA + and pA- RNA were separated from 10 μg HeLa nucleoplasm RNA using Dynabeads mRNA purification kit (Thermo Fisher) according to the manual. Ribosomal RNA were

depleted only from pA- RNA with Ribo-Zero rRNA depletion kit (Illumina). 500 ng of the isolated RNA were used to prepare the library.

Cell culture and in vivo splicing inhibition

Cell culture, siRNA transfection, in vivo splicing inhibition with Pla-B were as previously described (Nojima et al., 2015).

Empigen treatment in mNET-seq

Chromatin was isolated and digested with microccocal nuclease as previously described (Nojima et al., 2016; Nojima et al., 2015). EGTA (25 mM) was added to inactivate microccocal nuclease and digested chromatin was centrifuged at 13,000 rpm for 10 min to collect supernatant as soluble fraction. The soluble fraction was ten times diluted with NET-2 buffer (50 mM Tris-HCl pH 7.4, 150 mM NaCl and 0.05% NP-40) containing 1% empigen BB (Sigma) and added to Pol II antibody-conjugated beads for 1 hour immunoprecipitation. The beads were washed six times with NET-2 buffer containing 1% empigen BB. The rest of mNET-seq protocols were as previously described (Nojima et al., 2016).

Transcription unit annotation

hg19/GRCh37 was used as a reference genome. The matching ENSEMBL gene annotation (GRCh37.75) was used to extract transcription units (Flicek et al., 2014). This annotation was further complemented by transcription units annotated in NONCODE v4 (Xie et al., 2014), UCSC tRNA (Lowe and Eddy, 1997), PROMPT (Ntini et al., 2013) and eRNA (Andersson et al., 2014).

PROMPTs were extracted for the PROMPT genes in HeLa cells as previously annotated (Ntini et al., 2013). PROMPT 5’ ends were defined as the position within [TSS-3000, TSS+1000] with the maximal CAGE signal within EXOSC3 (hRrp40) KD environment. The PROMPT 3’ end was defined as the maximal 3’ TAG signal in EXOSC3 KD within the same region, provided it was downstream of the PROMPT 5’ end.

eRNA were taken from the PrESSTo database, part of the FANTOM5 project. Only ubiquitously expressed eRNA across cells and organs were used as they were most likely to also be present in HeLa cells (Andersson et al., 2014).

The ENSEMBL gene biotype annotation as defined by the second column in the gtf file was simplified into reduced categories (Table S1).

All Genes were taken from the most 5’ transcription start site (TSS) and most 3’ transcription end site (TES). Overlapping exons were merged to only include the most 5’ and most 3’ exon borders. Each gene was considered as not expressed (silent) if the interval [TSS, min (TSS+500,TES)] lacked sufficient signal in the chromatin fraction as well as the nucleoplasm fraction (defined by total number of reads mapping, fpkm and maximal signal within the interval).

Once silent regions were excluded, a further number of overlapping features on the same strand were also excluded under the following conditions: ENSEMBL lincRNA were excluded from further analysis if they overlapped coding genes (1 kbp extended at 5’ end and 3 kbp extended at 3’ end) or an antisense biotype labeled gene. Coding genes were excluded if they overlapped a lincRNA. lincRNA obtained from the NONCODE database were excluded if they overlapped an ENSEMBL lincRNA, eRNA, antisense RNA, ncRNA, another NONCODE annotation, coding gene, tRNA, pseudogene or PROMPT. eRNA were excluded if they overlapped an ENSEMBL lincRNA, pseudogene, ncRNA, tRNA, antisense RNA, PROMPT or coding gene. ncRNA were excluded if they overlapped a lincRNA, eRNA, another ncRNA, antisense RNA, tRNA, pseudogene, coding gene or PROMPT. tRNA were excluded if they overlapped an ENSEMBL lincRNA, eRNA, ncRNA, NONCODE lincRNA, tRNA, PROMPT, pseudogene, coding gene or antisense RNA. Antisense RNA were excluded if they overlapped an ENSEMBL lincRNA, eRNA, pseudogene, ncRNA, tRNA or PROMPT. PROMPTs were excluded if they overlapped an ENSEMBL lincRNA or a tRNA.

For the final list of used annotated regions, ENSEMBL lincRNA and NONCODE lincRNA were manually cross-checked with the siLuc chromomatin fraction RNA-seq to ensure that they are adequately expressed and do not fall into regions, which are likely to be read-through

from neighboring genes. This resulted in the selection of 285 lincRNA from both databases. TUs on chrY were not taken into consideration.

The above procedure generated a final feature annotation file, which was used for the majority of the analysis (Table S2, Dataset 1).

Data processing and presentation

mNET-Seq data and chromatin RNA-seq was processed as follows: mNET-Seq adapters were trimmed with Cutadapt v. 1.8.3 ((Martin, 2011), https://cutadapt. readthedocs.io/en/stable/) in paired end mode with the following parameters: -A GATCGTCGGACTGTAGAACTCTGAAC -a TGGAATTCTCGGGTGCCAAGG --minimum-length 10. Obtained sequences were mapped to the human hg19 reference sequence with Tophat v. 2.0.13 ((Kim et al., 2013), https://ccb.jhu.edu/software/tophat/) and the parameters -g 1 -r 3000 --no-coverage-search. Only properly paired and mapped reads were used for subsequent analysis (samflags 0x63, 0x93, 0x53, 0xA3), which were extracted with SAMtools v. 1.2 ((Li et al., 2009), http://www.htslib.org/). For mNET-Seq profiles only the most 3' nucleotide of the second read was used with the strandedness of the first read. Data was visualized with Bedtools v. 2.23.0 (genomeCoverageBed) ((Quinlan and Hall, 2010), http://www.htslib.org/). Trackhubs in the UCSC browser were created by employing the UCSC bedGraphToBigWig tool (Kent et al., 2002).

Metagene profiles

All profiles have averaged sense and antisense coverage around the indicated 5' or 3' sites of genomic features, except eRNA genes, where center of the annotated eRNA gene coordinates is taken as a reference point. As eRNA genes are bi-directional and therefore not associated with sense/antisense direction, the coverage-values for enhancers are shown on the plus and minus strand. For each metagene plot ≤1% of most extreme coverage-values in each bp-location were trimmed before averaging. For smoothing purposes data was binned into 10 bp

bins, error bars indicate the SEM across each bin. On rare occasions where an overlapping ncRNA caused large noise in the metagene profile, the corresponding TU was excluded from the analysis resulting in a small variation in considered number of lincRNA. Graphs were created using ggplot2 (http://www.ggplot2.org/) in R (http://www.R-project.org/).

Heatmaps

CTD profile heatmaps at the TSS and TES (Figure 1B) were generated by binning coding genes and lincRNA genes of length greater than 1000 nt into 100 bins. Profiles are shown with an additional 20 bins 5' of the TSS and 3' of the TES. Genes were ordered in descending order according to signal throughout the binned vector in each CTD phosphorylation isoform. Splicing associated S5P signal heatmaps at the 5'ss were generated by extracting the annotated exons for coding genes and lincRNA. Overlapping exons were grouped into one with the most extreme 5' and 3' boundaries. Only non-terminal exons were considered for the signal description of the 5'ss. Heatmaps were generated for bins of 10 bps within the [5'ss – 400 bp, 5'ss +400 bp] region. Genes were sorted according to the maximal signal of the bin with coordinates [5'ss -9bp, 5'ss] in the control sample and the same sorting was applied to the Pla-B treated sample. The S5P signal at the 5'ss was considered a peak if it was the maximal signal in the [5'ss -100bp, 5'ss +100bp] interval, allowing the computation of the proportion of peaks in coding vs. lincRNA exons (Figure 2D bottom). Heatmaps were created using the MATLAB R2015b (The MathWorks, Inc., Natick, MA, US) image function.

Escaping and termination indices

Escaping and Termination indices were computed on a single nucleotide basis from the mNET-seq profiles. In detail, the last nucleotide of the second read with the strandedness of the first read was formatted into bed format with the associated read names for all CTD phospho isoforms. These bedfiles were overlapped with [TSS, TSS+500], [TES, TES+2000] and GB (referring to gene body, defined as the middle 50% of the interval [TSS+500, TES]) using bedtools intersect with –s –c parameters. All counts were normalised to the length of

the corresponding region. The Escaping index EI was then defined as

$$EI = \log_2\left(\frac{\frac{[TSS, TSS+500nt]_{counts}}{500}}{\frac{GB_{counts}}{length_{GB}}}\right)$$

and the termination index TI was then defined as

$$TI = \log_2\left(\frac{\frac{[TES, TES+2000nt]_{counts}}{2000}}{\frac{GB_{counts}}{length_{GB}}}\right)$$

Splicing index

The Splicing index was computed from the nucleoplasm, nucleoplasm pA+ and nucleoplasm pA- fractions. Only exons, which are not annotated as the first exon were used and only the most extreme boundaries of overlapping exons were considered to compute this index. Spliced reads were extracted from sam files of all properly paired, properly mapped reads based on the CIGAR string containing the 'N'-label and mapped to the corresponding 3' and 5' splice sites. The number of spliced reads mapping to the 3'ss of the used exons were computed and defined as splicing events. Reads spanning the intron-exon junction were computed. In detail, the 2 nucleotides around the 3'SS were extracted (last nucleotide in the intron and first nucleotide in the exon). Reads overlapping these two nucleotides were computed with the bedtools intersect tool using the –f 1 option, i.e. enforcing the read to overlap both nucleotides. Only reads where the first mate is on the same strand as the 3'SS were considered. The splicing index was computed only for 3'SS which have non-zero levels of spliced reads at the site and with non-zero levels of reads spanning the intron-exon junction. The splicing index was defined as SI = (reads spliced at 3'SS) / (reads spanning 3'SS Intron-Exon junction) – hence the larger the SI the more efficient the splicing.

Ratio of nucleoplasm RNA and chromatin RNA and FPKM values

The ratio of nucleoplasm and chromatin RNA for siLuc and siEX3 data was computed with the R DESeq package (Anders and Huber, 2010). For each TU, the region [TSS, TSS+500] was overlapped with the RNA-seq reads using the inbuilt function summarizeOverlaps. FPKM values were computed with the same package using the estimateSizeFactors and fpkm functions.

pA ratio

The strand specific overlap of pA+ and pA- fragments with whole length coding and lincRNA genes was counted using bedtools intersect –c. The log2 ratio was taken of $pA-_{counts}/pA+_{counts}$ for each TU.

Principal Component Analysis

Principal Component Analysis was performed on the following ratios: chromatin siEX3/siLuc, nucleoplasmic pA-/pA+, nucleoplasm RNA/chromatin RNA, cytoplasm RNA/chromatin RNA (Mayer et al., 2015). Principal components were computed with the R prcomp function and the data was centered and scaled to zero mean and unit variance. Prcomp was applied to data of protein coding genes and the principal component rotation was subsequently applied to the data of lincRNA and antisense RNA. To identify lincRNA most similar to coding genes the cutoffs PC1>0 and PC2<1 were employed. Similarly for identification of coding genes most similar to lincRNA PC1<0 and PC2>1 were used. PCA was visualised with ggbiplot (http://github.com/vqv/ggbiplot).

P-values , significance tests and boxplots

P-values for Figure 2D (bottom) were computed with a Fisher Exact Test. All other p-values were computed by a Wilcoxon rank sum test in R. For Figure 4 we employed the paired Wilcoxon signed rank test. All boxplots were created with ggplot2 in R.

Scatterplots

Scatterplots were generated in R based on the FPKM values for the first 500nt of all analysed TUs. The Spearman correlation coefficient was computed in R. The identity line is indicated.

SUPPLEMENTARY REFERENCES

Anders, S., and Huber, W. (2010). Differential expression analysis for sequence count data. Genome biology *11*, R106.

Andersson, R., Gebhard, C., Miguel-Escalada, I., Hoof, I., Bornholdt, J., Boyd, M., Chen, Y., Zhao, X., Schmidl, C., Suzuki, T.*, et al.* (2014). An atlas of active enhancers across human cell types and tissues. Nature *507*, 455-461.

Dhir, A., Dhir, S., Proudfoot, N.J., and Jopling, C.L. (2015). Microprocessor mediates transcriptional termination of long noncoding RNA transcripts hosting microRNAs. Nat Struct Mol Biol *22*, 319-327.

Flicek, P., Amode, M.R., Barrell, D., Beal, K., Billis, K., Brent, S., Carvalho-Silva, D., Clapham, P., Coates, G., Fitzgerald, S.*, et al.* (2014). Ensembl 2014. Nucleic acids research *42*, D749-755.

Kent, W.J., Sugnet, C.W., Furey, T.S., Roskin, K.M., Pringle, T.H., Zahler, A.M., and Haussler, D. (2002). The human genome browser at UCSC. Genome research *12*, 996-1006.

Kim, D., Pertea, G., Trapnell, C., Pimentel, H., Kelley, R., and Salzberg, S.L. (2013). TopHat2: accurate alignment of transcriptomes in the presence of insertions, deletions and gene fusions. Genome biology *14*, R36.

Li, H., Handsaker, B., Wysoker, A., Fennell, T., Ruan, J., Homer, N., Marth, G., Abecasis, G., Durbin, R., and Genome Project Data Processing, S. (2009). The Sequence Alignment/Map format and SAMtools. Bioinformatics *25*, 2078-2079.

Lowe, T.M., and Eddy, S.R. (1997). tRNAscan-SE: a program for improved detection of transfer RNA genes in genomic sequence. Nucleic acids research *25*, 955-964.

Martin, M. (2011). Cutadapt removes adapter sequences from high-throughput sequencing reads. EMBnetjournal.

Mayer, A., di Iulio, J., Maleri, S., Eser, U., Vierstra, J., Reynolds, A., Sandstrom, R., Stamatoyannopoulos, J.A., and Churchman, L.S. (2015). Native elongating transcript sequencing reveals human transcriptional activity at nucleotide resolution. Cell *161*, 541-554.

Nojima, T., Gomes, T., Carmo-Fonseca, M., and Proudfoot, N.J. (2016). Mammalian NET-seq analysis defines nascent RNA profiles and associated RNA processing genome-wide. Nature protocols *11*, 413-428.

Nojima, T., Gomes, T., Grosso, A.R., Kimura, H., Dye, M.J., Dhir, S., Carmo-Fonseca, M., and Proudfoot, N.J. (2015). Mammalian NET-Seq Reveals Genome-wide Nascent Transcription Coupled to RNA Processing. Cell *161*, 526-540.

Ntini, E., Jarvelin, A.I., Bornholdt, J., Chen, Y., Boyd, M., Jorgensen, M., Andersson, R., Hoof, I., Schein, A., Andersen, P.R.*, et al.* (2013). Polyadenylation site-induced decay of upstream transcripts enforces promoter directionality. Nat Struct Mol Biol *20*, 923-928.

Quinlan, A.R., and Hall, I.M. (2010). BEDTools: a flexible suite of utilities for comparing genomic features. Bioinformatics *26*, 841-842.

Xie, C., Yuan, J., Li, H., Li, M., Zhao, G., Bu, D., Zhu, W., Wu, W., Chen, R., and Zhao, Y. (2014). NONCODEv4: exploring the world of long non-coding RNA genes. Nucleic acids research *42*, D98-103.
